# Association of Obesity With Prescription Opioids for Painful Conditions in Patients Seeking Primary Care in the US

**DOI:** 10.1001/jamanetworkopen.2020.2012

**Published:** 2020-04-02

**Authors:** Andrew Stokes, Dielle J. Lundberg, Bethany Sheridan, Katherine Hempstead, Natalia E. Morone, Karen E. Lasser, Ludovic Trinquart, Tuhina Neogi

**Affiliations:** 1 Department of Global Health, Boston University School of Public Health, Boston, Massachusetts; 2 athenahealth Inc, Watertown, Massachusetts; 3 Robert Wood Johnson Foundation, Princeton, New Jersey; 4 Section of General Internal Medicine, Boston University School of Medicine, Boston, Massachusetts; 5 Department of Community Health Sciences, Boston University School of Public Health, Boston, Massachusetts; 6 Department of Biostatistics, Boston University School of Public Health, Boston, Massachusetts; 7 Section of Rheumatology, Boston University School of Medicine, Boston, Massachusetts; 8 Department of Epidemiology, Boston University School of Public Health, Boston, Massachusetts

## Abstract

**Importance:**

Prior studies have identified an association between obesity and prescription opioid use in the US. However, the pain conditions that are factors in this association remain unestablished.

**Objective:**

To investigate the association between obesity and pain diagnoses recorded by primary care clinicians as reasons for prescription of opioids.

**Design, Setting, and Participants:**

A cross-sectional study including 565 930 patients aged 35 to 64 years with a body mass index (BMI) measurement recorded in 2016 was conducted. Electronic health records of patients seen by primary care clinicians in the US in the multipayer athenahealth network from January 1, 2015, to December 31, 2017, were reviewed, and data were analyzed from March 1 to September 15, 2019.

**Main Outcomes and Measures:**

Any prescription of opioids in the 365 days before or after the first BMI measurement in 2016 were identified. All *International Classification of Diseases, Ninth Revision*, and *International Statistical Classification of Diseases and Related Health Problems, Tenth Revision*, claims within 7 days before each opioid prescription were captured and classified using a pain diagnosis typologic system. Weight was categorized as underweight (BMI, 18.5-19.9), normal weight (BMI, 20.0-24.9), overweight (BMI, 25.0-29.9), obese I (BMI, 30-34.9), obese II (BMI, 35.0-39.9), obese III (BMI, 40.0-49.9), and obese IV (BMI, 50.0-80.0).

**Results:**

Among 565 930 patients, 329 083 (58.1%) were women. A total of 125 093 patients (22.1%) were aged 35 to 44 years, 199 384 patients (35.2%) were 45 to 54 years, and 241 453 patients (42.7%) were 55 to 64 years. A total of 177 631 patients (31.4%) were overweight and 273 135 patients (48.2%) were obese at baseline. Over 2 years, 93 954 patients (16.6%) were prescribed opioids. The risk of receiving prescription opioids increased progressively with BMI (adjusted relative risk for overweight: 1.08; 95% CI, 1.06-1.10; obese I: 1.24; 95% CI, 1.22-1.26; obese II: 1.33; 95% CI, 1.30-1.36; obese III: 1.48; 95% CI, 1.45-1.51; and obese IV, 1.71; 95% CI, 1.65-1.77). The percentage of patients with opioid prescriptions attributable to an overweight or obese BMI was 16.2% (95% CI, 15.0%-17.4%). Prescription opioids for management of osteoarthritis (relative risk for obese vs normal weight, 1.90; 95% CI, 1.77-2.05) and other joint disorders (relative risk, 1.63; 95% CI, 1.55-1.72) both had stronger associations with obesity than the mean for any pain diagnosis (relative risk, 1.33; 95% CI, 1.31-1.36). Osteoarthritis, other joint disorders, and other back disorders comprised a combined 53.4% of the absolute difference in prescription of opioids by obesity.

**Conclusions and Relevance:**

Joint and back disorders appear to be the most important diagnoses in explaining the increased receipt of opioid prescriptions among patients with obesity. Addressing the opioid crisis will require attention to underlying sources of demand for prescription opioids, including obesity, through its associations with pain.

## Key Points

**Question:**

Is obesity associated with prescription opioids for pain diagnoses among patients seeking primary care in the United States?

**Findings:**

In this cross-sectional study of electronic health records of 565 930 patients, osteoarthritis, other joint disorders, and other back disorders were the most important pain diagnoses associated with the increased prescribing of opioids to patients with obesity.

**Meaning:**

The findings suggest that population-level efforts to prevent and treat obesity may reduce the prevalence and severity of joint and back disorders and the associated use of prescription opioids.

## Teaser

This cross-sectional study investigates the association of obesity with prescription opioids for pain diagnoses among US adults seeking primary care.

## Introduction

Since the late 1990s, opioid prescriptions have more than tripled in the US.^1^ In 2015, 91 million adults reported prescription opioid use.^2^ Among these adults, 12.5% reported prescription opioid misuse, and most adults who misused opioids described relief of physical pain as a primary motivation.

Considerable evidence exists that supply-side factors, including aggressive marketing of opioids by pharmaceutical companies; new standards of care in long-term, noncancer pain management; and increased patient expectations for pain relief are all factors in the dramatic increase in prescription opioid use.^3,4,5,6^ Other researchers have posited that trends in chronic pain, depression, and socioeconomic despair related to stagnant wages, job loss, and declining social status have increased the demand for prescription opioids among patients.^7,8,9,10,11,12,13^

Another potential demand-side factor that has received less attention is obesity. Obesity has a strong positive association with chronic pain through a physiologic mechanism of increasing the risk and severity of painful conditions, such as arthritis and low back pain.^14,15,16,17^ A psychosocial pathway also exists via depression and disrupted sleep, which may lead to fatigue, inactivity, decreased mobility, and amplification of pain through pain sensitization.^18,19,20,21^

A recent analysis of the National Health and Nutrition Examination Survey found evidence of a cross-sectional association between obesity and prescription opioid use.^22^ However, the specific pain diagnoses underlying this association were not well established. Identifying the pain conditions associated with the obesity–prescription opioid association is an important step toward developing targeted interventions to reach patients who are most likely to receive opioids and inform the prioritization of pain management efforts nationwide.

In this study, we used multipayer electronic health record data from the athenahealth network to investigate the association between obesity and prescription opioids. We then examined the association of obesity with prescription of opioids for specific pain diagnoses. Next, we evaluated the diagnoses most associated with the overall difference in prescription of opioids by obesity. In addition, we explored variations in these associations and the prevalence of specific pain diagnoses by sociodemographic traits.

## Methods

### Data Source

Athenahealth Inc is a commercial vendor and developer of cloud-based practice management and electronic health record software for a network of outpatient primary care practices.^23^ The national network includes more than 60 million patients seeking care annually from more than 120 000 health professionals in the US.^24^

Our analysis relied on deidentified electronic health record data from the athenahealth network. The Boston University Medical Campus Institutional Review Board determined that this analysis of deidentified data qualified as exempt human participants research. This study followed the Strengthening the Reporting of Observational Studies in Epidemiology (STROBE) reporting guideline.

 This study included patients in the athenahealth network from January 1, 2015, to December 31, 2017, with at least 1 body mass index (BMI) (calculated as weight in kilograms divided by height in meters squared) reading taken during a visit with a primary care clinician, such as a physician with a specialty in internal medicine, family medicine, or general practice; a nurse practitioner; or a physician assistant, in 2016. We limited the sample to patients aged 35 to 64 years as of January 1, 2016. To ensure that we only included patients with sufficient data on their history and who remained in the network for the study’s duration, we limited the sample to patients who had at least 1 primary care visit more than 2 years before their first BMI reading in 2016 and at least 1 primary care visit more than 1 year after. Patients with a cancer or pregnancy diagnosis within 1 year of their first BMI reading, recorded an extreme BMI less than 18.5 or greater than 80, or had missing values for race/ethnicity or urbanicity were also excluded. These exclusions are detailed in eFigure 1 in the Supplement to yield a sample of 565 930 patients.

### Study Design and Measures

In this cross-sectional study, a patient’s first BMI reading by their primary care clinician in 2016 served as the index value. Prescriptions written by a primary care clinician during the 365 days before or after the index date were captured to determine whether the patient received any opioid prescriptions. Depending on the patient’s index date, the earliest opioid prescription could have occurred on January 1, 2015, and the latest could have occurred December 31, 2017. We captured all diagnosis claims within 7 days before each opioid prescription.

Body mass index was measured by the patient’s primary care clinician and was categorized as underweight (BMI, 18.5-19.9), normal weight (BMI, 20.0-24.9), overweight (BMI, 25.0-29.9), obese I (BMI, 30.0-34.9), obese II (BMI, 35.0-39.9), obese III (BMI, 40.0-49.9), and obese IV (BMI, 50.0-80.0).^25^ Other covariates included age (35-39, 40-44, 45-49, 50-54, 55-59, and 60-64 years), sex (male, female), race/ethnicity (non-Hispanic white, non-Hispanic black, Hispanic, non-Hispanic other), census region (Northeast, Midwest, South, and West), urbanicity (metropolitan, metro-adjacent, and rural), and health insurance (commercial, Medicaid, Medicare, and other).

### Prescription Opioids

eTable 3 in the Supplement presents a list of 13 opioid analgesic medications included in our definition of prescription opioids. Opioid antitussives and medication-assisted treatments for opioid use disorders were excluded.

We used a typologic system developed by Sherry et al^26^ to generate a list of *International Classification of Diseases, Ninth Revision,* codes for conditions that commonly cause pain severe enough to require opioid prescriptions. The list was reviewed by a rheumatologist (T.N.) and internal medicine physician (N.E.M.), who categorized the codes into 25 distinct pain diagnoses. eTable 4 and eTable 5 in the Supplement present the *International Classification of Diseases, Ninth Revision*, and *International Statistical Classification of Diseases and Related Health Problems, Tenth Revision*, codes used to classify the diagnoses.

We documented whether patients received prescription opioids for management of each of the 25 diagnoses. Patients could have more than 1 diagnosis if they had multiple claims in the 7 days before an opioid prescription or if they had multiple prescriptions. Some patients had no pain diagnosis claim. In these instances, we classified diagnoses as none or other. One possible explanation for this phenomenon is that some patients were diagnosed with a painful condition before the study but renewed their prescriptions by telephone without an associated visit. Claims could have also been generated at a hospital or specialty clinic, but the prescription was written by the primary care clinician. In addition, the claim could have been recorded outside the 7-day window.

### Statistical Analysis

We used negative binomial regression with robust SEs to examine the association of BMI category with prescription of opioids, adjusting for age, sex, race/ethnicity, region, urbanicity, and health insurance status. To examine the sensitivity of the results to the exclusion of missing data, we used multiple imputation by chained equations to account for missing data on race/ethnicity and urbanicity and repeated the regression. We then estimated the fraction of patients whose prescription opioids could have been avoided under the hypothetical scenario in which patients who were overweight or obese were of normal weight. We determined the population-attributable fraction for each BMI category by calculating the observed number of cases in each category and the expected number of cases under exposure to the normal BMI category via marginal prediction.^27^

Next, we assessed the association of obesity with prescription of opioids for management of each pain diagnosis separately. Based on these models, we estimated the marginal percentages of patients receiving prescription opioids overall and for each diagnosis among patients with obesity and patients with normal weight. Using these estimates, we determined the absolute difference in the percentage of patients with prescription opioids, comparing patients with obesity with those of normal weight. We repeated this calculation to find the absolute difference in prescription opioids for each specific pain diagnosis. In addition, by dividing the absolute difference for each diagnosis by the absolute difference for prescription opioids overall, we determined the percentage contribution of each diagnosis to the overall difference in patients with prescription opioids by obesity.

To assess whether patients reported pain diagnoses alone or in combinations, we examined a subsample of patients with obesity who received prescription opioids and had 1 or more pain diagnoses. We tabulated the unadjusted percentage of patients who recorded each of the 15 most common diagnoses alone and in combination with each of the other 14 most common diagnoses.

In addition, we stratified the sample by each covariate, using negative binomial regression models to calculate the strata-specific association of obesity and any prescription opioids, adjusting for all other covariates. We then estimated the marginal prevalence of each diagnosis among patients with obesity in the strata and ranked the prevalence on a scale from 1 to 25 to explore relative differences between strata. Two-tailed *P* values less than .05 calculated with an unpaired *t* test were considered statistically significant. We conducted all analyses using Stata, version 15 (StataCorp), from March 1 to September 15, 2019.

## Results

The sample included 565 930 patients aged 35 to 64 years (35-44 years, 125 093 [22.1%]; 45-54 years, 199 384 [35.2%]; and 55-64 years, 241 453 [42.7%]). Of these, 329 083 patients (58.1%) were women and 455 923 patients (80.6%) were non-Hispanic white. At baseline, 177 631 patients (31.4%) were overweight and 273 135 patients (48.2%) were obese. Over 2 years, 93 954 patients (16.6%) were prescribed opioids (Table 1). Among those with prescription opioids, 79 109 patients (84.2%) had a pain diagnosis claim in the 7 days before their opioid prescription.

**Table 1.** Baseline Demographic Characteristics of Patients Aged 35 to 64 Years in the athenahealth Network, 2015-2017^a^

Variable	No. (%)		Patients with prescription opioids, unadjusted %
	Total sample (N = 565 930)	Patients with prescription opioids (n = 93 954)	
Sex			
Men	236 847 (41.9)	38 451 (40.9)	16.2
Women	329 083 (58.1)	55 503 (59.1)	16.9
Age, y			
35-39	55 254 (9.8)	7541 (8.0)	13.6
40-44	69 839 (12.3)	9915 (10.6)	14.2
45-49	89 088 (15.7)	13 870 (14.8)	15.6
50-54	110 296 (19.5)	19 032 (20.3)	17.3
55-59	121 958 (21.6)	22 177 (23.6)	18.2
60-64	119 495 (21.1)	21 419 (22.8)	17.9
Race/ethnicity			
Non-Hispanic			
White	455 923 (80.6)	75 861 (80.7)	16.6
Black	49 419 (8.7)	9970 (10.6)	20.2
Non-Hispanic other	20 357 (3.6)	1798 (1.9)	8.8
Hispanic	40 231 (7.1)	6325 (6.7)	15.7
Urban/rural			
Metropolitan	462 338 (81.7)	69 682 (74.2)	15.1
Metro-adjacent	71 880 (12.7)	17 428 (18.5)	24.2
Rural	31 712 (5.6)	6844 (7.3)	21.6
Census region			
Northeast	190 992 (33.7)	18 511 (19.7)	9.7
Midwest	72 612 (12.8)	13 279 (14.1)	18.3
South	255 799 (45.2)	53 283 (56.7)	20.8
West	46 527 (8.2)	8881 (9.5)	19.1
Health insurance			
Commercial	437 848 (77.4)	55 710 (59.3)	12.7
Medicaid	46 409 (8.2)	12 613 (13.4)	27.2
Medicare	57 886 (10.2)	20 044 (21.3)	34.6
Other	23 787 (4.2)	5587 (5.9)	23.5
BMI^b^			
Underweight	10 486 (1.9)	1720 (1.8)	16.4
Normal weight	104 678 (18.5)	13 608 (14.5)	13.0
Overweight	177 631 (31.4)	25 580 (27.2)	14.4
Obese			
I	139 499 (24.6)	24 216 (25.8)	17.4
II	74 002 (13.1)	14 514 (15.4)	19.6
III	49 464 (8.7)	11 318 (12.0)	22.9
IV	10 170 (1.8)	2998 (3.2)	29.5

Abbreviation: BMI, body mass index (calculated as weight in kilograms divided by height in meters squared).

^a^ Baseline characteristics recorded at patient's first primary care visit during 2016.

^b^ Underweight: BMI, 18.5 to 19.9; normal weight: 20.0 to 24.9; overweight: 25.0 to 29.9; obese I: 30.0 to 34.9; obese II: 35.0 to 39.9; obese III: 40.0 to 49.9; and obese IV: 50.0 to 80.0.

The risk of receiving prescription opioids increased progressively with BMI (adjusted relative risk [RR] for overweight: 1.08; 95% CI, 1.06-1.10; obese I: 1.24; 95% CI, 1.22-1.26; obese II: 1.33; 95% CI, 1.30-1.36; obese III: 1.48; 95% CI, 1.45-1.51; and obese IV: 1.71; 95% CI, 1.65-1.77). The complete regression results are presented in eTable 1 in the Supplement. The association between BMI and prescription opioids was similar in a sensitivity analysis using multiply imputed data (adjusted RR for overweight: 1.09; 95% CI, 1.07-1.11; obese I: 1.25; 95% CI, 1.23-1.27; obese II: 1.35; 95% CI, 1.32-1.38; obese III: 1.51; 95% CI, 1.47-1.54; and obese IV: 1.74; 95% CI, 1.68, 1.80) (eTable 2 in the Supplement). At the population level, we estimated that the percentage of patients with prescription opioids attributable to having an overweight or obese BMI was 16.2% (95% CI, 15.0%-17.4%) (Table 2).

**Table 2.** Relative Risks and PAFs for Receiving Prescription Opioids by BMI Category in 565 930 Patients Aged 35 to 64 Years, athenahealth Network 2015 to 2017^a^

BMI category^b^	Relative risk (95% CI)^c^^,^^d^	PAF, % (95% CI)^c^
Underweight	1.15 (1.10-1.21)	0.2 (0.2-0.3)
Normal weight	1 [Reference]	
Overweight	1.08 (1.06-1.10)	2.1 (1.6-2.6)
Obese		
I	1.24 (1.22-1.26)	5.0 (4.6-5.4)
II	1.33 (1.30-1.36)	3.9 (3.6-4.2)
III	1.48 (1.45-1.51)	3.9 (3.7-4.2)
IV	1.71 (1.65-1.77)	1.3 (1.2-1.5)
Total (overweight to obese IV)	NA	16.2 (15.0-17.4)

Abbreviations: BMI, body mass index (calculated as weight in kilograms divided by height in meters squared); NA, not applicable; PAF, population-attributable fraction.

^a^ eTable 1 in the Supplement provides the complete regression results. Sample interpretation: 5.0% of patients receiving prescription opioids could be avoided in the hypothetical situation in which those with a BMI of obese I had a normal BMI.

^b^ Underweight: BMI, 18.5 to 19.9; normal weight: 20.0 to 24.9; overweight: 25.0 to 29.9; obese I: 30.0 to 34.9; obese II: 35.0 to 39.9; obese III: 40.0 to 49.9; and obese IV: 50.0 to 80.0.

^c^ Adjusted for age, sex, race/ethnicity, region, urbanicity, and health insurance.

^d^ Relative risk calculated using a negative binomial model with robust SEs.

**Figure 1.**
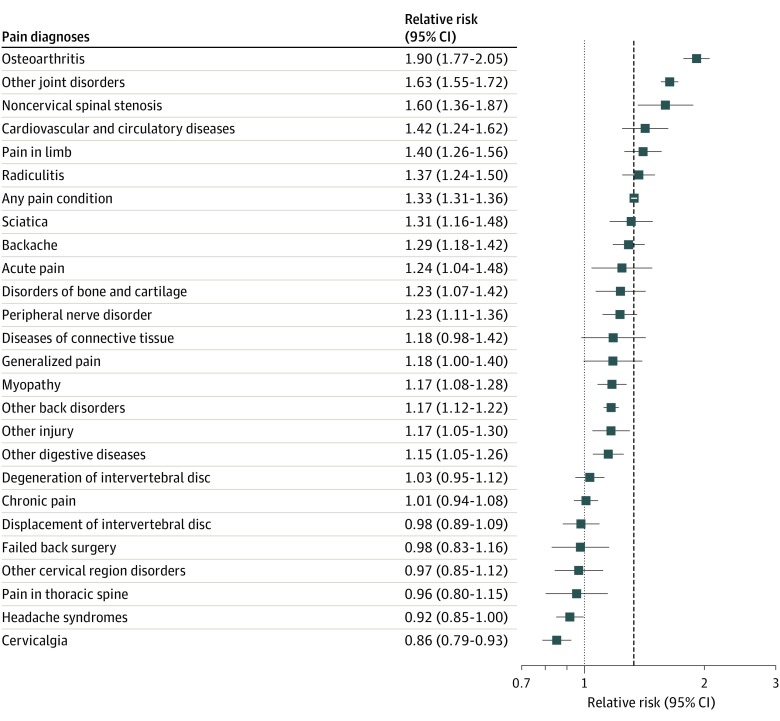
Relative Risks of Receiving Prescription Opioids for Specific Pain Diagnoses by Obesity (Obese vs Normal Weight) Among 555 444 Patients Aged 35 to 64 Years, athenahealth Network, 2015-2017 Obesity was positively associated with risk of receiving prescription opioids for 16 pain diagnoses. Osteoarthritis and other joint disorders were the 2 reasons for receiving prescription opioids that were most strongly associated with obesity. The dashed line represents the overall relative risk of receiving prescription opioids for any pain diagnosis by obesity.

Figure 1 presents the RR of patients receiving opioid prescriptions for management of specific pain conditions, comparing patients with obesity with patients with normal weight (n = 555 444). Sixteen pain diagnoses recorded with prescription opioids had a significant positive association with obesity. Prescription opioids for osteoarthritis (RR for obese vs normal weight, 1.90; 95% CI, 1.77-2.05), other joint disorders (eg, arthropathies, reactive arthritis, joint dislocations, and other pain in joints) (RR, 1.63; 95% CI, 1.55-1.72), and noncervical spinal stenosis (RR, 1.60; 95% CI, 1.36-1.87) all had stronger associations than the mean for any pain diagnosis (RR, 1.33; 95% CI, 1.31-1.36). Patients with obesity had an RR of 1.90 (95% CI, 1.77-2.05) for receiving prescription opioids for management of osteoarthritis and 1.63 (95% CI, 1.55-1.72) for receiving opioids for management of other joint disorders compared with patients with normal weight.

The absolute difference in prevalence of prescription opioids between patients with obesity and patients with normal weight was 4.6% (95% CI, 4.3%-4.9%). Several pain diagnoses emerged as factors in this difference. Other joint disorders (26.6%), osteoarthritis (16.9%), and other back disorders (eg, spondylosis and low back pain) (9.9%) were responsible for a combined 53.4% of the elevated prevalence of any prescription opioids in patients with obesity. Radiculitis (4.6%), backache (4.0%), and pain in limb (3.9%) emerged as lesser but still positive factors. Headache syndromes (−1.5%) and cervicalgia (−2.6%) were both less common in patients with obesity and thus were negatively associated with the difference in prescription opioids by obesity (Table 3).

**Table 3.** Prevalence and Absolute Differences in Patients With Prescription Opioids for Management of Specific Pain Diagnoses by Obesity Among 555 444 Patients Aged 35 to 64 Years, athenahealth Network, 2015-2017^a^

Pain diagnosis recorded with prescription opioids^b^	Prevalence, % (95% CI)			Obese vs normal	
	Overall	Normal (BMI 20.0-24.9)	Obese (BMI>30)	Absolute differences, % (95% CI)	Contribution to overall differences, %
Any pain condition	16.6 (16.5-16.7)	13.9 (13.7-14.1)	18.5 (18.3-18.6)	4.6 (4.3-4.9)	100
Other joint disorders	2.7 (2.6-2.7)	1.9 (1.8-2.0)	3.2 (3.1-3.2)	1.2 (1.1-1.3)	26.6
Osteoarthritis	1.3 (1.3-1.4)	0.86 (0.80-0.92)	1.6 (1.6-1.7)	0.78 (0.70-0.85)	16.9
Other back disorders	3.0 (3.0-3.1)	2.7 (2.6-2.8)	3.2 (3.1-3.2)	0.46 (0.34-0.58)	9.9
Radiculitis	0.72 (0.70-0.74)	0.58 (0.53-0.63)	0.80 (0.77-0.83)	0.21 (0.16-0.27)	4.6
Backache	0.74 (0.72-0.76)	0.63 (0.58-0.68)	0.81 (0.78-0.85)	0.18 (0.12-0.25)	4.0
Pain in limb	0.56 (0.54-0.58)	0.45 (0.41-0.49)	0.63 (0.60-0.66)	0.18 (0.13-0.23)	3.9
Myopathy	0.79 (0.77-0.81)	0.71 (0.66-0.76)	0.84 (0.80-0.87)	0.12 (0.06-0.19)	2.7
Peripheral nerve disorder	0.60 (0.58-0.62)	0.54 (0.49-0.58)	0.66 (0.63-0.69)	0.12 (0.07-0.18)	2.7
Cardiovascular and circulatory diseases	0.36 (0.34-0.37)	0.29 (0.25-0.32)	0.41 (0.38-0.43)	0.12 (0.08-0.16)	2.6
Noncervical spinal stenosis	0.27 (0.26-0.29)	0.20 (0.17-0.23)	0.32 (0.30-0.34)	0.12 (0.08-0.16)	2.6
Sciatica	0.42 (0.40-0.44)	0.34 (0.31-0.38)	0.45 (0.42-0.47)	0.11 (0.06-0.15)	2.3
Other digestive diseases	0.73 (0.71-0.75)	0.67 (0.62-0.72)	0.76 (0.73-0.80)	0.10 (0.04-0.16)	2.2
Other injury	0.49 (0.47-0.51)	0.45 (0.41-0.49)	0.53 (0.50-0.55)	0.08 (0.03-0.13)	1.6
Disorders of bone and cartilage	0.29 (0.28-0.30)	0.25 (0.22-0.28)	0.31 (0.29-0.33)	0.06 (0.02-0.10)	1.3
Acute pain	0.20 (0.19-0.21)	0.18 (0.15-0.20)	0.22 (0.20-0.24)	0.04 (0.01-0.08)	0.9
Generalized pain	0.21 (0.20-0.22)	0.19 (0.16-0.22)	0.22 (0.21-0.24)	0.03 (0.00-0.07)	0.7
Diseases of connective tissue	0.17 (0.16-0.18)	0.15 (0.13-0.18)	0.18 (0.17-0.20)	0.03 (0.00-0.06)	0.6
Degeneration of intervertebral disc	0.80 (0.78-0.82)	0.79 (0.73-0.85)	0.82 (0.78-0.85)	0.03 (−0.04 to 0.09)	0.6
Chronic pain	1.1 (1.1-1.2)	1.1 (1.1-1.2)	1.2 (1.1-1.2)	0.01 (−0.07 to 0.09)	0.3
Failed back surgery	0.20 (0.19-0.22)	0.20 (0.17-0.23)	0.20 (0.18-0.22)	0.00 (−0.04 to 0.03)	−0.1
Pain in thoracic spine	0.16 (0.15-0.17)	0.17 (0.15-0.20)	0.17 (0.15-0.18)	−0.01 (−0.04 to 0.02)	−0.2
Other cervical region disorders	0.28 (0.27-0.30)	0.29 (0.25-0.32)	0.28 (0.26-0.30)	−0.01 (−0.05 to 0.03)	−0.2
Displacement of intervertebral disc	0.50 (0.48 to 0.52)	0.51 (0.46 to 0.55)	0.50 (0.47 to 0.52)	−0.01 (−0.06 to 0.05)	−0.2
Headache syndromes	0.82 (0.79 to 0.84)	0.86 (0.81 to 0.92)	0.80 (0.76 to 0.83)	−0.07 (−0.13 to 0.00)	−1.5
Cervicalgia	0.75 (0.73 to 0.77)	0.83 (0.77 to 0.88)	0.71 (0.68 to 0.74)	−0.12 (−0.19 to −0.05)	−2.6

Abbreviation: BMI, body mass index (calculated as weight in kilograms divided by height in meters squared).

^a^ Adjusted for age, sex, race/ethnicity, region, urbanicity, and health insurance. Patients with underweight BMI (18.5-19.9) were excluded.

^b^ eTable 4 and eTable 5 in the Supplement provide *International Classification of Diseases, Ninth Revision*, and *International Statistical Classification of Diseases and Related Health Problems, Tenth Revision*, codes used to diagnose each pain diagnosis. Patients could record multiple pain diagnosis claims prior to receiving prescription opioids. A total of 14 845 patients (15.8%) with prescription opioids also recorded no diagnosis claim within 7 days prior to prescription.

**Figure 2.**
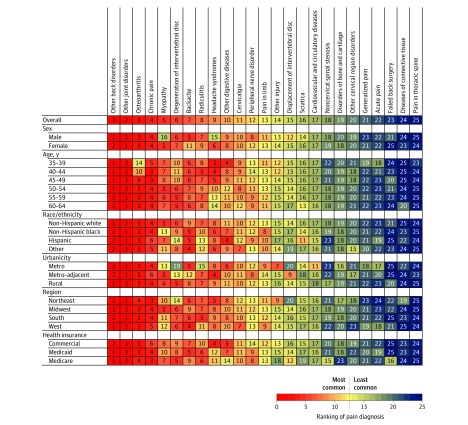
Ranking of Pain Diagnoses Reported With Prescription Opioids Among 273 135 Patients With Obesity Aged 35 to 64 Years Stratified by Sociodemographic Traits, athenahealth Network 2015-2017 Adjusted for age, sex, race/ethnicity, region, urbanicity, and health insurance. Patients with obesity had a body mass index above 30 (calculated as weight in kilograms divided by height in meters squared).

Figure 2 presents the ranked prevalence of pain diagnoses reported with prescription opioids among patients with obesity (n = 273 135). Other back disorders, other joint disorders, and osteoarthritis were the 3 most common diagnoses. After stratifying to explore differences by sex, age, race/ethnicity, urbanicity, region, and health insurance, other back disorders and other joint disorders remained the first and second most prevalent diagnoses across all strata. Osteoarthritis was ranked third or fourth in all but 2 strata, which were the younger age strata at which osteoarthritis does not typically occur. Approximately 30% of patients who recorded a pain diagnosis with prescription opioids recorded more than 1 diagnosis. The 2 most common combinations were other back disorders with other joint disorders (3.3%) and other back disorders with radiculitis (2.7%) (eFigure 2 in the Supplement).

The association of BMI with prescription opioids was stronger among women (RR, 1.43; 95% CI, 1.40-1.46) compared with men (RR, 1.15; 95% CI, 1.12-1.19); in the Northeast (RR, 1.64; 95% CI, 1.57-1.70) compared with the Midwest (RR, 1.18; 95% CI, 1.13-1.24), South (RR, 1.23; 95% CI, 1.20-1.26), and West (RR, 1.41; 95% CI, 1.33-1.49]); and in metropolitan areas (RR, 1.40; 95% CI, 1.37-1.43) compared with metro-adjacent (RR, 1.11; 95% CI, 1.07-1.16) and rural (RR, 1.13; 95% CI, 1.06-1.20) areas. (eFigure 3 in the Supplement). Among patients with obesity who received prescription opioids, myopathy and headache syndromes were relatively more common among women, and the backache diagnosis was more common among men. Osteoarthritis and degeneration of the intervertebral disc were both more prevalent among older patients. Headache syndromes, in contrast, were more common in patients younger than 50 years. Myopathy, degeneration of the intervertebral disc, and radiculitis were all less common in metropolitan areas, and degeneration of the intervertebral disc was less common in the Northeast (Figure 2).

## Discussion

In this cross-sectional study of a national sample of electronic health records, we found a significant association between obesity and prescription opioids that increased with progressively higher BMI. Patients in the obese III category had a 48% increased risk of receiving prescription opioids and patients in the obese IV category had a 71% increased risk. At the population level, we estimated that overweight and obese BMI accounted for 16.2% of patients receiving opioid prescriptions.

The obesity–prescription opioid association was largely explained by 3 pain diagnoses—osteoarthritis, other joint disorders, and other back disorders—that comprised 53.4% of the absolute difference in risk of prescription opioids by obesity. These 3 diagnoses were also the most prevalent diagnoses overall, and osteoarthritis and other joint disorders were the 2 reasons for prescription opioids most closely associated with obesity. Prescription opioids for other back disorders were less strongly associated with obesity but remained a significant factor because of their high prevalence.

Obesity is an established risk factor for osteoarthritis, which most commonly affects the lower limbs.^28^ The pathogenesis of osteoarthritis is related to obesity through excessive joint loading, altered biomechanics, inflammation related to adipose tissue, and potential hormonal dysregulation.^16,29,30^ Thus, osteoarthritis may cause pain in weight-bearing and non–weight-bearing joints.^31,32^ Obesity duration may also increase the risk of osteoarthritis.^33,34^ Our results suggest that osteoarthritis was a particularly important factor in the risk of receiving prescription opioids among patients with obesity older than 45 years. Many of the conditions captured in the other joint pain diagnoses could also reflect undiagnosed arthritis.^35^

Obesity is further associated with several back disorders through a similar set of biomechanical and inflammatory pathways.^17,36^ Receiving prescription opioids for management of intervertebral disc degeneration was not as strongly associated with obesity as prescription opioids for other back disorders. One possible explanation for this finding is that, depending on the location of the affected intervertebral discs, degenerative disc disease could manifest as back pain or as neck pain, and the latter is not as strongly affected by excessive loading caused by weight gain.^36^

Moving forward, our findings suggest a need for greater attention to the prevention and management of painful conditions that underlie the demand for prescription opioids, in particular, joint and back disorders. Reducing prescription opioid use will require expanding access to integrated pain treatments, including nonopioid medications, and nonpharmacologic approaches, such as physical therapy, cognitive behavioral therapy, and acupuncture.^37,38^ These solutions will be particularly important given more restrictive opioid-prescribing guidelines, which may increase the number of patients living with unmanaged chronic pain.^39,40^ Tailored physical activity and nutritional counseling interventions, medications for weight loss, and bariatric surgery to reduce obesity could also help reduce pain severity and prevent disability.^15,41,42,43^ Because environmental factors across the life course may reduce individual capacity to regulate personal diet and physical activity, health policies are also needed to change the obesogenic environment.^44^ Such efforts may include nutritional labeling, advertising restrictions, targeted taxes or subsidies, public awareness campaigns, regulation of nutritional quality in school cafeterias, and programs to increase the availability and affordability of healthy foods.^45^

Greater awareness is also needed throughout the public health community on how the burden of obesity at the population level includes chronic pain, and in this way, how obesity has factored in to the increased demand for prescription opioids in the US. Specifically, joint and back pain appear to have played a significant role in increasing the demand for pain management among patients, which manufacturers and health care professionals responded to with prescription opioids.^46,47^ Thus, future investments in primary and secondary obesity prevention could be framed as efforts to reduce the burden of chronic pain, prescription opioid use, and ultimately future societal costs of the opioid epidemic, including opioid-related overdose and mortality.^44,48^

### Limitations

This study has several limitations. First, owing to the study’s cross-sectional design, it is not possible to ascertain causality in the associations between obesity, prescription opioids, and pain diagnosis outcomes. We also cannot rule out residual confounding by smoking status, depression, socioeconomic status, and other variables not available in the deidentified data we received from athenahealth. Second, all prescription opioid data in this study refer to prescriptions written, which may not have been filled or, if the prescriptions were filled, the patients also may not have used the medications. Third, patients who seek health care are less likely to be healthy than adults in the general population; thus, associations observed in this study could be weaker than at the population level. Fourth, 15.8% of patients with prescription opioids had no associated pain diagnosis in the electronic health record system during the 7-day window before their opioid prescription. As a result, we may have underestimated our reported prevalence values. Fifth, we were unable to examine obesity duration in this study.

## Conclusions

In this cross-sectional study of patients seeking primary care in the US from 2015 to 2017, BMI was associated with prescription of opioids. Osteoarthritis, other joint disorders, and other back disorders were the most common pain diagnoses observed in patients with prescription opioids and appeared to be factors most associated with opioid prescriptions in patients with obesity. Future research should address the specific pathways between obesity, chronic pain, the increased demand for prescription opioids, and the epidemic of opioid misuse in the US. As the prevalence of obesity continues to increase, this study provides data for policymakers to consider greater investments in primary and secondary obesity prevention within a comprehensive response to the opioid epidemic.

## Notes

Supplement. **eTable 1.** Negative Binomial Regression of BMI and Any Prescription Opioids (n=565,930) **eTable 2.** Negative Binomial Regression of BMI and Any Prescription Opioids Using Multiply Imputed Data (n=632,535) **eFigure 1.** Sample Inclusion/Exclusion Criteria **eFigure 2.** Percent Recording a Pain Diagnosis Alone or in Combination With a Second Diagnosis Among Patients With Obesity Aged 35-64 With 1+ Prescription Opioid and Diagnosis, athenahealth Network, 2015-2017 (n=44,747) **eFigure 3.** Relative Risk of Prescription Opioids by Obesity (Obese vs Normal Weight) in Subgroups Stratified by Sociodemographic Characteristics (n=555,444) **eTable 3.** Opioid Analgesic Medications Identified as Prescription Opioids **eTable 4.** ICD-9 Typology to Identify Pain Diagnosis Claims **eTable 5.** ICD-10 Crosswalk From ICD-9 Typology to Identify Pain Diagnosis Claims

## References

[1] Guy GP Jr, Zhang K, Bohm MK, Vital signs: changes in opioid prescribing in the United States, 2006-2015. MMWR Morb Mortal Wkly Rep. 2017;66(26):-. doi:10.15585/mmwr.mm6626a4 28683056 PMC5726238

[2] Han B, Compton WM, Blanco C, Crane E, Lee J, Jones CM Prescription opioid use, misuse, and use disorders in US adults: 2015 national survey on drug use and health. Ann Intern Med. 2017;167(5):293-301. doi:10.7326/M17-0865 28761945

[3] Dasgupta N, Beletsky L, Ciccarone D Opioid crisis: no easy fix to its social and economic determinants. Am J Public Health. 2018;108(2):182-186. doi:10.2105/AJPH.2017.304187 29267060 PMC5846593

[4] Van Zee A The promotion and marketing of oxycontin: commercial triumph, public health tragedy. Am J Public Health. 2009;99(2):221-227. doi:10.2105/AJPH.2007.131714 18799767 PMC2622774

[5] Hadland SE, Rivera-Aguirre A, Marshall BDL, Cerdá M Association of pharmaceutical industry marketing of opioid products with mortality from opioid-related overdoses. JAMA Netw Open. 2019;2(1):e186007. doi:10.1001/jamanetworkopen.2018.6007 30657529 PMC6484875

[6] Kaye AD, Jones MR, Kaye AM, Prescription opioid abuse in chronic pain: an updated review of opioid abuse predictors and strategies to curb opioid abuse: part 1. Pain Physician. 2017;20(2S):S93-S109. doi:10.36076/ppj.2017.s111 28226333

[7] Currie J, Jin JY, Schnell M US employment and opioids: is there a connection? National Bureau of Economic Research; working paper 24440. Updated April 2019. Accessed September 1, 2019. https://www.nber.org/papers/w24440.pdf

[8] Graham C, Pinto S Unequal hopes and lives in the USA: optimism, race, place, and premature mortality. J Popul Econ. 2019;32(2):665-733. doi:10.1007/s00148-018-0687-y

[9] Hollingsworth A, Ruhm C, Simon K Macroeconomic Conditions and Opioid Abuse. Vol 56 Elsevier; 2017. doi:10.3386/w23192 29128677

[10] Ruhm C Deaths of Despair or Drug Problems? National Bureau of Economic Research; 2018. doi:10.3386/w24188

[11] Case A, Deaton A Rising morbidity and mortality in midlife among white non-Hispanic Americans in the 21st century. Proc Natl Acad Sci U S A. 2015;112(49):15078-15083. doi:10.1073/pnas.1518393112 26575631 PMC4679063

[12] Glei DA, Stokes A, Weinstein M Changes in mental health, pain, and drug misuse since the mid-1990s: is there a link? Soc Sci Med. 2020;246:112789. doi:10.1016/j.socscimed.2020.112789 31978637 PMC7064160

[13] Venkataramani AS, Bair EF, O’Brien RL, Tsai AC Association between automotive assembly plant closures and opioid overdose mortality in the United States: a difference-in-differences analysis. JAMA Intern Med. 2019;180(2):254-262. doi:10.1001/jamainternmed.2019.5686 31886844 PMC6990761

[14] Shiri R, Falah-Hassani K, Heliövaara M, Risk factors for low back pain: a population-based longitudinal study. Arthritis Care Res (Hoboken). 2019;71(2):290-299. doi:10.1002/acr.23710 30044543

[15] Dunlop DD, Song J, Semanik PA, Relation of physical activity time to incident disability in community dwelling adults with or at risk of knee arthritis: prospective cohort study. BMJ. 2014;348(4):g2472. doi:10.1136/bmj.g2472 24782514 PMC4004786

[16] King LK, March L, Anandacoomarasamy A Obesity & osteoarthritis. Indian J Med Res. 2013;138(2):185-193. 24056594 PMC3788203

[17] Shiri R, Karppinen J, Leino-Arjas P, Solovieva S, Viikari-Juntura E The association between obesity and low back pain: a meta-analysis. Am J Epidemiol. 2010;171(2):135-154. doi:10.1093/aje/kwp356 20007994

[18] Institute of Medicine Relieving Pain in America: A Blueprint for Transforming Prevention, Care, Education, and Research. National Academies Press; 2011. doi:10.17226/13172. 22553896

[19] Okifuji A, Donaldson GW, Barck L, Fine PG Relationship between fibromyalgia and obesity in pain, function, mood, and sleep. J Pain. 2010;11(12):1329-1337. doi:10.1016/j.jpain.2010.03.006 20542742 PMC2939916

[20] Jantaratnotai N, Mosikanon K, Lee Y, McIntyre RS The interface of depression and obesity. Obes Res Clin Pract. 2017;11(1):1-10. doi:10.1016/j.orcp.2016.07.003 27498907

[21] Katz P, Margaretten M, Trupin L, Schmajuk G, Yazdany J, Yelin E Role of sleep disturbance, depression, obesity, and physical inactivity in fatigue in rheumatoid arthritis. Arthritis Care Res (Hoboken). 2016;68(1):81-90. doi:10.1002/acr.22577 25779719 PMC6083443

[22] Stokes A, Berry KM, Collins JM, The contribution of obesity to prescription opioid use in the United States. Pain. 2019;160(10):2255-2262. doi:10.1097/j.pain.0000000000001612 31149978 PMC6756256

[23] Hempstead K, Sung I, Gray J, Richardson S Tracking trends in provider reimbursements and patient obligations. Health Aff (Millwood). 2015;34(7):1220-1224. doi:10.1377/hlthaff.2015.0105 26153318

[24] athenahealth. The athenahealth network. Published 2020. Accessed February 27, 2020. https://www.athenahealth.com/why-choose-us/healthcare-network

[25] Organization WH Obesity: Preventing and Managing the Global Epidemic. World Health Organization; 2000. 11234459

[26] Sherry TB, Sabety A, Maestas N Documented pain diagnoses in adults prescribed opioids: results from the National Ambulatory Medical Care Survey, 2006-2015. Ann Intern Med. 2018;169(12):892-894. doi:10.7326/M18-0644 30208400 PMC6296869

[27] Mansournia MA, Altman DG Population attributable fraction. BMJ. 2018;360:k757. doi:10.1136/bmj.k757 29472187

[28] Kulkarni K, Karssiens T, Kumar V, Pandit H Obesity and osteoarthritis. Maturitas. 2016;89:22-28. doi:10.1016/j.maturitas.2016.04.006 27180156

[29] Urban H, Little CB The role of fat and inflammation in the pathogenesis and management of osteoarthritis. Rheumatology (Oxford). 2018;57(suppl_4):iv10-iv21. doi:10.1093/rheumatology/kex399 29444323

[30] Collins KH, Sharif B, Reimer RA, Association of Metabolic markers with self-reported osteoarthritis among middle-aged BMI-defined non-obese individuals: a cross-sectional study. BMC Obes. 2018;5:23. doi:10.1186/s40608-018-0201-9 30186613 PMC6120068

[31] Koonce RC, Bravman JT Obesity and osteoarthritis: more than just wear and tear. J Am Acad Orthop Surg. 2013;21(3):161-169. doi:10.5435/JAAOS-21-03-161 23457066

[32] Vuolteenaho K, Koskinen A, Moilanen E Leptin—a link between obesity and osteoarthritis. applications for prevention and treatment. Basic Clin Pharmacol Toxicol. 2014;114(1):103-108. doi:10.1111/bcpt.12160 24138453

[33] Guss JD, Ziemian SN, Luna M, The effects of metabolic syndrome, obesity, and the gut microbiome on load-induced osteoarthritis. Osteoarthritis Cartilage. 2019;27(1):129-139. doi:10.1016/j.joca.2018.07.020 30240938 PMC6309743

[34] Gushue DL, Houck J, Lerner AL Effects of childhood obesity on three-dimensional knee joint biomechanics during walking. J Pediatr Orthop. 2005;25(6):763-768. doi:10.1097/01.bpo.0000176163.17098.f4 16294133

[35] Guglielmo D, Murphy LB, Boring MA, State-specific severe joint pain and physical inactivity among adults with arthritis—United States, 2017. MMWR Morb Mortal Wkly Rep. 2019;68(17):381-387. doi:10.15585/mmwr.mm6817a2 31048678 PMC6541316

[36] Peng T, Pérez A, Pettee Gabriel K The Association among overweight, obesity, and low back pain in u.s. adults: a cross-sectional study of the 2015 National Health Interview Survey. J Manipulative Physiol Ther. 2018;41(4):294-303. doi:10.1016/j.jmpt.2017.10.005 29459122

[37] Tick H, Nielsen A, Pelletier KR, ; Pain Task Force of the Academic Consortium for Integrative Medicine and Health Evidence-based nonpharmacologic strategies for comprehensive pain care: the Consortium Pain Task Force white paper. Explore (NY). 2018;14(3):177-211. doi:10.1016/j.explore.2018.02.001 29735382

[38] Herman PM, Lavelle TA, Sorbero ME, Hurwitz EL, Coulter ID Are nonpharmacologic interventions for chronic low back pain more cost effective than usual care? proof of concept results from a Markov model. Spine (Phila Pa 1976). 2019;44(20):1456-1464. doi:10.1097/BRS.0000000000003097 31095119 PMC6779140

[39] Pergolizzi JV Jr, Rosenblatt M, LeQuang JA Three years down the road: the aftermath of the CDC guideline for prescribing opioids for chronic pain. Adv Ther. 2019;36(6):1235-1240. doi:10.1007/s12325-019-00954-1 31016474 PMC6824381

[40] Stokes A, Berry KM, Hempstead K, Lundberg DJ, Neogi T Trends in prescription analgesic use among adults with musculoskeletal conditions in the United States, 1999-2016. JAMA Netw Open. 2019;2(12):e1917228. doi:10.1001/jamanetworkopen.2019.17228 31825504 PMC6991204

[41] Messier SP, Mihalko SL, Legault C, Effects of intensive diet and exercise on knee joint loads, inflammation, and clinical outcomes among overweight and obese adults with knee osteoarthritis: the IDEA randomized clinical trial. JAMA. 2013;310(12):1263-1273. doi:10.1001/jama.2013.277669 24065013 PMC4450354

[42] White DK, Neogi T, Rejeski WJ, ; Look AHEAD Research Group Can an intensive diet and exercise program prevent knee pain among overweight adults at high risk? Arthritis Care Res (Hoboken). 2015;67(7):965-971. doi:10.1002/acr.22544 25692781 PMC4482772

[43] King WC, Chen J-Y, Belle SH, Change in pain and physical function following bariatric surgery for severe obesity. JAMA. 2016;315(13):1362-1371. doi:10.1001/jama.2016.3010 27046364 PMC4856477

[44] Gortmaker SL, Swinburn BA, Levy D, Changing the future of obesity: science, policy, and action. Lancet. 2011;378(9793):838-847. doi:10.1016/S0140-6736(11)60815-5 21872752 PMC3417037

[45] Hawkes C, Jewell J, Allen K A food policy package for healthy diets and the prevention of obesity and diet-related non-communicable diseases: the NOURISHING framework. Obes Rev. 2013;14(suppl 2):159-168. doi:10.1111/obr.12098 24103073

[46] Maly A, Vallerand AH Neighborhood, socioeconomic, and racial influence on chronic pain. Pain Manag Nurs. 2018;19(1):14-22. doi:10.1016/j.pmn.2017.11.004 29422123 PMC8895435

[47] Sites BD, Harrison J, Herrick MD, Masaracchia MM, Beach ML, Davis MA Prescription opioid use and satisfaction with care among adults with musculoskeletal conditions. Ann Fam Med. 2018;16(1):6-13. doi:10.1370/afm.2148 29311169 PMC5758314

[48] Swinburn BA, Sacks G, Hall KD, The global obesity pandemic: shaped by global drivers and local environments. Lancet. 2011;378(9793):804-814. doi:10.1016/S0140-6736(11)60813-1 21872749

