# Supplementary material for: Association of Obesity With Prescription Opioids for Painful Conditions in Patients Seeking Primary Care in the US
Source: JAMA Netw Open. 2020 Apr 2;3(4):e202012. doi: 10.1001/jamanetworkopen.2020.2012 (PMC7118518; doi:10.1001/jamanetworkopen.2020.2012)
Supplement: Supplement. — eTable 1. Negative Binomial Regression of BMI and Any Prescription Opioids (n=565,930) eTable 2. Negative Binomial Regression of BMI and Any Prescription Opioids Using Multiply Imputed Data (n=632,535) eFigure 1. Sample Inclusion/Exclusion Criteria eFigure 2. Percent Recording a Pain Diagnosis Alone or in Combination With a Second Diagnosis Among Patients With Obesity Aged 35-64 With 1+ Prescription Opioid and Diagnosis, athenahealth Network, 2015-2017 (n=44,747) eFigure 3. Relative Risk of Prescription Opioids by Obesity (Obese vs Normal Weight) in Subgroups Stratified by Sociodemographic Characteristics (n=555,444) eTable 3. Opioid Analgesic Medications Identified as Prescription Opioids eTable 4. ICD-9 Typology to Identify Pain Diagnosis Claims eTable 5. ICD-10 Crosswalk From ICD-9 Typology to Identify Pain Diagnosis Claims [file jamanetwopen-3-e202012-s001.pdf]

Supplementary Online Content

Stokes A, Lundberg DJ, Sheridan B, et al. Association of obesity with prescription opioids for painful conditions in patients seeking primary care in the US. *JAMA Netw Open*. 2020;3(4):e202012. doi:10.1001/jamanetworkopen.2020.2012

eTable 1. Negative Binomial Regression of BMI and Any Prescription Opioids (n=565,930)
eTable 2. Negative Binomial Regression of BMI and Any Prescription Opioids Using Multiply Imputed Data (n=632,535)
eFigure 1. Sample Inclusion/Exclusion Criteria
eFigure 2. Percent Recording a Pain Diagnosis Alone or in Combination With a Second Diagnosis Among Patients With Obesity Aged 35-64 With 1+ Prescription Opioid and Diagnosis, athenahealth Network, 2015-2017 (n=44,747)
eFigure 3. Relative Risk of Prescription Opioids by Obesity (Obese vs Normal Weight) in Subgroups Stratified by Sociodemographic Characteristics (n=555,444)
eTable 3. Opioid Analgesic Medications Identified as Prescription Opioids
eTable 4. ICD-9 Typology to Identify Pain Diagnosis Claims
eTable 5. ICD-10 Crosswalk From ICD-9 Typology to Identify Pain Diagnosis Claims

This supplementary material has been provided by the authors to give readers additional information about their work.

© 2020 Stokes A et al. *JAMA Network Open.*

eTable 1. Negative binomial regression of BMI and any prescription opioids (n=565,930)

	Relative Risk	95% CI			P-Value
Body Mass Index (kg/m^2)					
Underweight (18.5 - 19.9)	1.15	1.10	,	1.21	<0.001
Normal Weight (20 - 24.9)	ref		-		-
Overweight (25 - 29.9)	1.08	1.06	,	1.10	<0.001
Obese I (30 - 34.9)	1.24	1.22	,	1.26	<0.001
Obese II (35 - 39.9)	1.33	1.30	,	1.36	<0.001
Obese III (40 - 49.9)	1.48	1.45	,	1.51	<0.001
Obese IV+ (50 - 80)	1.71	1.65	,	1.77	<0.001
Sex					
Male	ref		-		-
Female	1.03	1.02	,	1.05	<0.001
Age					
35-39	ref		-		-
40-44	1.06	1.03	,	1.09	<0.001
45-49	1.16	1.13	,	1.19	<0.001
50-54	1.25	1.22	,	1.28	<0.001
55-59	1.28	1.25	,	1.31	<0.001
60-64	1.17	1.15	,	1.20	<0.001
Race/Ethnicity					
Non-Hispanic White	ref		-		-
Non-Hispanic Black	0.91	0.89	,	0.92	<0.001
Hispanic	0.88	0.86	,	0.90	<0.001
Other	0.71	0.68	,	0.75	<0.001
Urban/Rural					
Metro	ref		-		-
Metro-Adjacent	1.27	1.25	,	1.29	<0.001
Rural	1.09	1.07	,	1.12	<0.001
Census Region					
Northeast	ref		-		-
Midwest	1.79	1.75	,	1.83	<0.001
South	1.88	1.85	,	1.91	<0.001
West	1.81	1.77	,	1.85	<0.001
Health Insurance					
Commercial	ref		-		-
Medicaid	2.15	2.11	,	2.19	<0.001
Medicare	2.40	2.36	,	2.43	<0.001
Other	1.62	1.58	,	1.66	<0.001

© 2020 Stokes A et al. *JAMA Network Open.*

eTable 2. Negative binomial regression of BMI and any prescription opioids using multiply imputed data (n=632,535)

	Relative Risk	95% CI			P-Value
Body Mass Index (kg/m^2)					
Underweight (18.5 - 19.9)	1.14	1.09	,	1.19	<0.001
Normal Weight (20 - 24.9)	ref		-		-
Overweight (25 - 29.9)	1.09	1.07	,	1.11	<0.001
Obese I (30 - 34.9)	1.25	1.23	,	1.27	<0.001
Obese II (35 - 39.9)	1.35	1.32	,	1.38	<0.001
Obese III (40 - 49.9)	1.51	1.47	,	1.54	<0.001
Obese IV+ (50 - 80)	1.74	1.68	,	1.80	<0.001
Sex					
Male	ref		-		-
Female	1.03	1.02	,	1.04	<0.001
Age					
35-39	ref		-		-
40-44					
45-49	1.08	1.05	,	1.11	<0.001
50-54	1.19	1.16	,	1.22	<0.001
55-59	1.29	1.26	,	1.33	<0.001
60-64	1.32	1.29	,	1.35	<0.001
Race/Ethnicity					
Non-Hispanic White	ref		-		-
Non-Hispanic Black	0.91	0.90	,	0.93	<0.001
Hispanic	0.87	0.85	,	0.89	<0.001
Other	0.71	0.68	,	0.74	<0.001
Urban/Rural					
Metro	ref		-		-
Metro-Adjacent	1.31	1.29	,	1.33	<0.001
Rural	1.14	1.11	,	1.16	<0.001
Census Region					
Northeast	ref		-		-
Midwest	1.78	1.75	,	1.82	<0.001
South	1.81	1.78	,	1.84	<0.001
West	1.76	1.72	,	1.80	<0.001
Health Insurance					
Commercial	ref		-		-
Medicaid	2.21	2.17	,	2.25	<0.001
Medicare	2.47	2.43	,	2.50	<0.001
Other	1.67	1.63	,	1.71	<0.001

a. Multiple imputation by chained equations was used to impute missing values of race/ethnicity and urbanicity.

b. Supplemental Table 1 presents the same regression in a sample where observations with missing values were excluded.

© 2020 Stokes A et al. *JAMA Network Open.*

eFigure 1. Sample inclusion/exclusion criteria

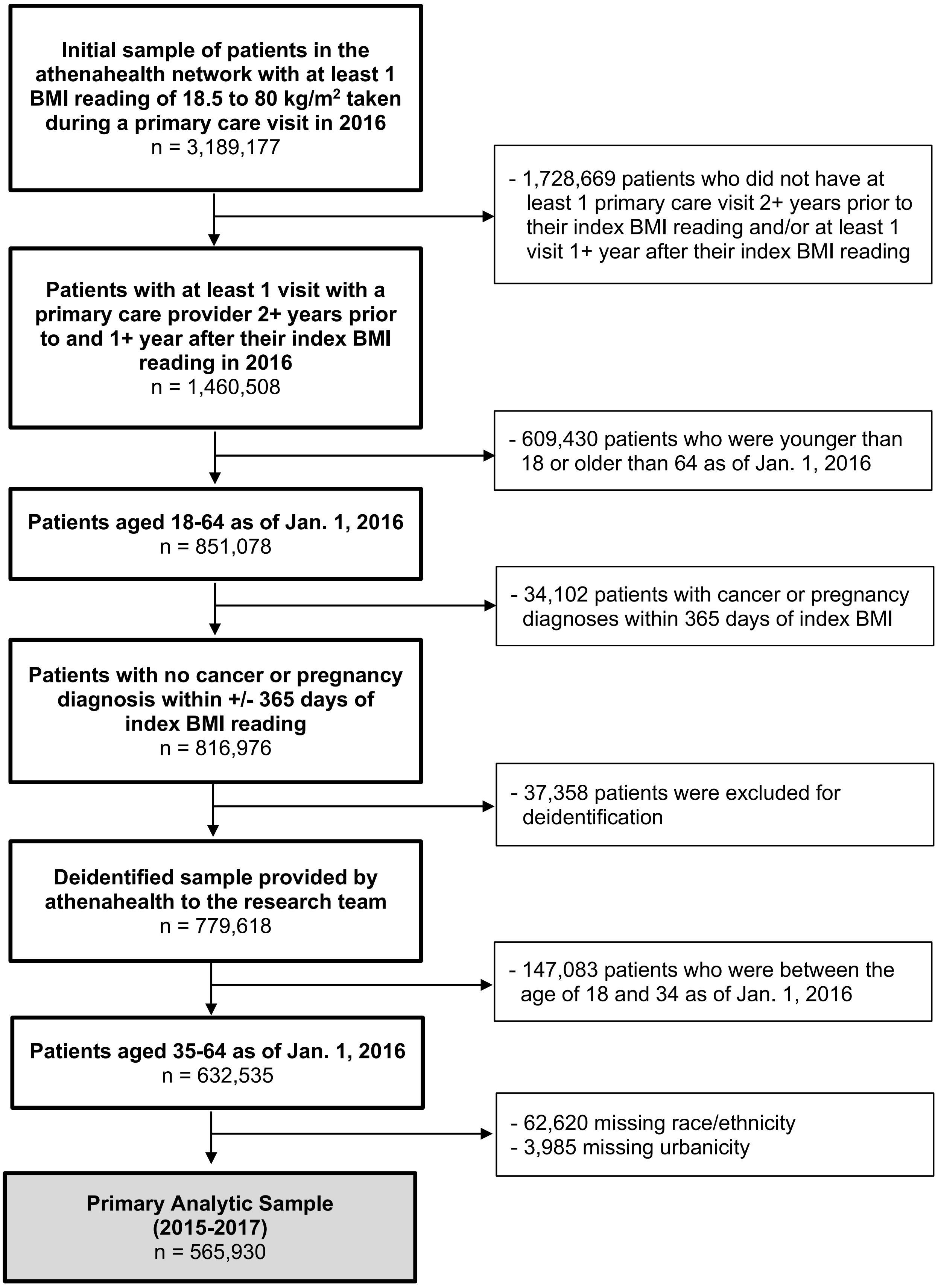

© 2020 Stokes A et al. *JAMA Network Open.*

eFigure 2. Percent recording a pain diagnosis alone or in combination with a second diagnosis among patients with obesity aged 35-64 with 1+ prescription opioid and diagnosis, athenahealth network, 2015-2017 (n=44,747)[a,b,c,d]

Other back disorders	Other joint disorders	Osteoarthritis	Chronic pain	Myopathy	Degeneration of disc	Backache	Radiculitis	Headache syndromes	Other digestive diseases	Cervicalgia	Peripheral nerve disorder	Pain in limb	Other injury	Displacement of disc	
8.6%	3.3%	1.8%	2.1%	1.9%	2.0%	0.48%	2.7%	0.65%	0.57%	1.5%	0.97%	0.63%	0.29%	1.1%	Other back disorders
	10.3%	2.2%	1.13%	1.21%	0.89%	0.56%	0.86%	0.52%	0.48%	0.97%	0.78%	0.93%	0.43%	0.51%	Other joint disorders
		4.8%	0.71%	1.00%	0.58%	0.21%	0.71%	0.26%	0.19%	0.29%	0.65%	0.25%	0.10%	0.28%	Osteoarthritis
			2.9%	0.54%	0.70%	0.27%	0.85%	0.25%	0.20%	0.51%	0.43%	0.23%	0.08%	0.38%	Chronic pain
				1.1%	0.50%	0.17%	0.86%	0.36%	0.15%	0.53%	0.46%	0.18%	0.06%	0.32%	Myopathy
					1.5%	0.17%	0.81%	0.19%	0.08%	0.49%	0.27%	0.14%	0.06%	0.51%	Degeneration of disc
						2.9%	0.13%	0.17%	0.20%	0.21%	0.14%	0.12%	0.11%	0.08%	Backache
							1.1%	0.15%	0.07%	0.41%	0.29%	0.16%	0.06%	0.48%	Radiculitis
								2.5%	0.22%	0.42%	0.17%	0.10%	0.10%	0.13%	Headache syndromes
									2.8%	0.08%	0.11%	0.08%	0.04%	0.06%	Other digestive diseases
										1.3%	0.27%	0.22%	0.10%	0.25%	Cervicalgia
											1.2%	0.23%	0.07%	0.22%	Peripheral nerve disorder
												1.8%	0.19%	0.09%	Pain in limb
													1.9%	0.05%	Other injury
														1.0%	Displacement of disc

a. Patients with obesity had a BMI above 30 kg/m^2.
b. Prevalence of each diagnosis or combination of diagnoses is unadjusted.
c. 15.8% of patients with prescription opioids in the sample recorded no pain diagnosis and were excluded.
d. See Appendix Tables 2 and 3 for ICD-9 and ICD-10 used to diagnose each pain condition.

© 2020 Stokes A et al. *JAMA Network Open.*

eFigure 3. Relative risk of prescription opioids by obesity (obese vs. normal weight) in subgroups stratified by sociodemographic characteristics (n=555,444)

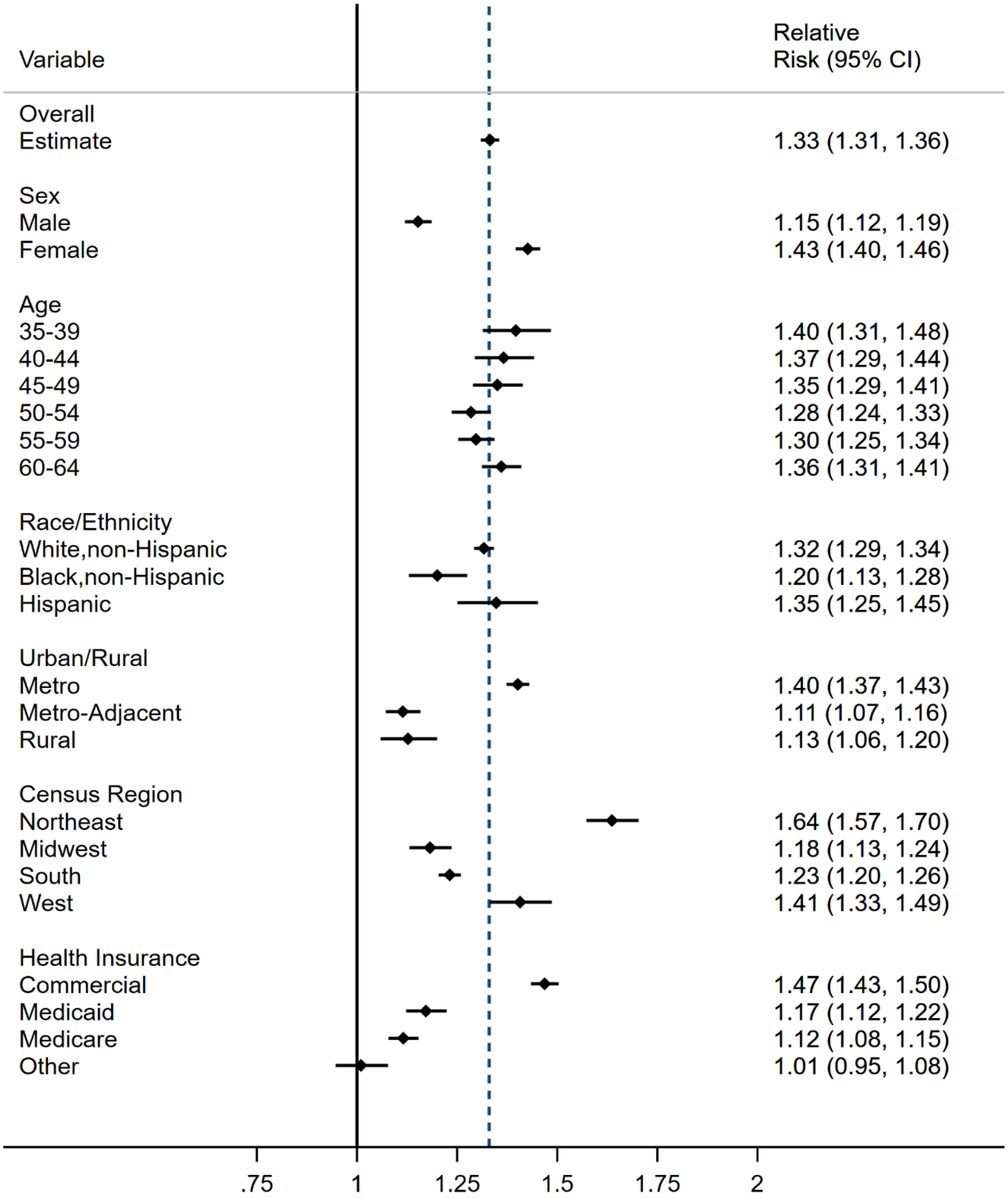

a. Patients with obesity had BMI above 30 kg/m^2. Patients with normal weight had BMI 20-24.9 kg/m^2. Patients who were underweight (18.5 to 19.9 kg/m^2) were excluded.

b. Relative risks were calculated using a negative binomial regression of BMI and prescription opioid use, adjusting for all covariates – age, sex, race/ethnicity, region, urbanicity, and health insurance – except for the covariate used to stratify.

c. Sample interpretation: female patients who were obese (30+ kg/m^2) were 1.43 times more likely to receive prescription opioids than female patients with normal BMI (20-24.9 kg/m^2). However, male patients who were obese were only 1.15 times more likely to receive prescription opioids than male patients with normal BMI.

© 2020 Stokes A et al. *JAMA Network Open.*

eTable 3. Opioid analgesic medications identified as prescription opioids

Medication category	Medication name
Opioid analgesic medications included in the study's definition of prescription opioids	Fentanyl
	Dihydrocodeine with pain reliever
	Codeine / dihydrocodeine / hydrocodone with pain reliever
	Morphine
	Oxycodone
	Tramadol
	Meperedine
	Hydromorphone
	Tapentadol
	Levorphanol
	Nalbuphine
	Oxymorphone
	Pentazocine
Opioid antitussives and medication-assisted treatments for opioid use disorders excluded from the study's definition of prescription opioids	Methadone
	Codeine / dihydrocodeine / hydrocodone with cough medicine
	Buprenorphine
	Suboxone
	Naloxone
	Ondansetron
	Naltrexone

© 2020 Stokes A et al. *JAMA Network Open.*

eTable 4. ICD-9 typology to identify pain diagnosis claims

Pain Diagnosis Claims	ICD-9 Code	Description of Code
Acute pain	338.1	Acute pain
Backache	724.5	Backache, unspecified
	724.8	Other symptoms referable to back
Cardiovascular and circulatory diseases	136.1	Behcet's syndrome
	393	Chronic rheumatic pericarditis
	415.1	Pulmonary embolism and infarction
	420	Acute pericarditis
	422	Acute myocarditis
	429	Myocarditis, unspecified
	444.2	Embolism and thrombosis of arteries of the extremities.
	444.8	Embolism and thrombosis of other specified artery
	444.9	Embolism and thrombosis of unspecified artery.
	445	Atheroembolism
	447.6	Arteritis, unspecified
	449	Septic arterial embolism
	451	Phlebitis and thrombophlebitis
	453	Budd-chiari syndrome
	453.1	Thrombophlebitis migrans
	453.4	Acute venous embolism and thrombosis of deep vessels of lower extremity
	453.82	Acute deep venous thrombosis (DVT) of left arm vein
	453.83	Acute venous embolism and thrombosis of upper extremity, unspecified
	453.84	Acute venous embolism and thrombosis of axillary veins
	453.89	Acute venous embolism and thrombosis of other specified veins
	454	Varicose veins of lower extremities with ulcer
	454.1	Varicose veins of lower extremities with inflammation
	454.2	Varicose veins of lower extremities with ulcer and inflammation
	454.8	Varicose veins of lower extremities with other complications
	457.1	Other lymphedema
	457.2	Lymphangitis
	786.5	Chest pain
Cervicalgia	723.1	Cervicalgia
	723.5	Torticollis, unspecified
Chronic pain	338.2	Chronic pain
	338.4	Chronic pain syndrome
Degeneration of intervetebral disc	722.4	Other cervical disc degeneration, unspecified cervical region
	722.5	Degeneration of thoracic or lumbar intervertebral disc
	722.6	Degeneration of intervertebral disc, site unspecified
Diseases of connective tissue	710.0	Systemic lupus erythematosus
	710.1	Systemic sclerosis
	723.6	Panniculitis specified as affecting neck
	727	Synovitis and tenosynovitis
	727.2	Specific bursitides often of occupational origin
	727.3	Other bursitis
	727.6	Rupture of tendon nontraumatic

© 2020 Stokes A et al. *JAMA Network Open.*

	729.3	Panniculitis unspecified
	729.4	Fasciitis, unspecified
Disorders of joint	099.3	Reactive arthritis
	713	Arthropathy associated with other disorders classified elsewhere
	716	Other and unspecified arthropathies
	717	Internal derangement of knee
	718	Articular cartilage disorder
	718.1	Loose body in joint
	718.2	Pathological dislocation
	718.3	Recurrent dislocation of joint
	718.8	Other joint derangement not elsewhere classified
	718.9	Unspecified derangement of joint
	719.1	Hemarthrosis
	719.2	Villonodular synovitis
	719.3	Palindromic rheumatism
	719.4	Pain in joint
	729	Rheumatism, unspecified and fibrositis
	274.0	Gouty arthropathy
	274.9	Gout, unspecified.
	712	Crystal arthropathies
	714.0	Rheumatoid arthritis, unspecified
	714.1	Felty's syndrome
	714.2	Other rheumatoid arthritis with visceral or systemic involvement
	714.3	Juvenile chronic polyarthritis
	714.4	Chronic postrheumatic arthropathy
	714.89	Other specified inflammatory polyarthropathies
	714.9	Unspecified inflammatory polyarthropathy
	696	Psoriasis and similar disorders
	720	Ankylosing spondylitis and other inflammatory spondylopathies
Disorders of bone and cartilage	588.0	Renal osteodystrophy
	724.6	Disorders of sacrum
	724.7	Disorders of coccyx
	731.2	Hypertrophic pulmonary osteoarthropathy
	733.1	Pathologic fracture
	733.4	Aseptic necrosis of bone
	733.93	Stress fracture of tibia or fibula
	733.94	Stress fracture, unspecified toe(s), initial encounter for fracture
	733.95	Stress fracture of other bone
	733.96	Stress fracture of femoral neck
	733.97	Stress fracture of shaft of femur
	733.98	Stress fracture of pelvis
	726.0	Adhesive capsulitis of unspecified shoulder.
	726.1	Rotator cuff syndrome of shoulder and allied disorders.
	726.2	Other affections of shoulder region, not elsewhere classified
	726.3	Enthesopathy of elbow region
	726.4	Enthesopathy of wrist and carpus

© 2020 Stokes A et al. *JAMA Network Open.*

	726.5	Enthesopathy of hip region
	726.6	Enthesopathy of knee
	726.7	Enthesopathy of ankle and tarsus.
	726.8	Other peripheral enthesopathies
	726.9	Unspecified enthesopathy
Displacement of intervertebral disc	722.0	Displacement of cervical intervertebral disc without myelopathy
	722.1	Displacement of thoracic or lumbar intervertebral disc without myelopathy
	722.2	Unspecified thoracic, thoracolumbar and lumbosacral intervertebral disc disorder
	722.3	Schmorl's nodes
Generalized pain, symptoms	780.96	Pain, unspecified
Headache syndromes	339	Other headache syndromes
	784	Headache
	346	Migraine
Myopathy	359.4	Toxic myopathy
	359.5	Myopathy in endocrine diseases classified elsewhere
	359.6	Symptomatic inflammatory myopathy in diseases classified elsewhere
	359.7	Inflammatory and immune myopathies, not elsewhere classified
	359.8	Other myopathies
	359.9	Myopathy, unspecified
	710.3	Dermatomyositis
	710.4	Polymyositis
	710.5	Eosinophilia myalgia syndrome
	725	Polymyalgia rheumatica
	728.1	Muscular calcification and ossification
	728.81	Interstitial myositis
	728.83	Rupture of muscle, nontraumatic
	728.85	Spasm of muscle
	728.88	Rhabdomyolysis
	729.1	Myalgia and myositis, unspecified
	729.7	Nontraumatic compartment syndrome
	729.82	Cramp and spasm
Osteoarthritis	715	Osteoarthrosis and allied disorders
Other back disorders	724.2	Lumbago
	724.9	Other unspecified back disorders
	721	Spondylosis and allied disorders
Other cervical region disorders	723.2	Cervicocranial syndrome
	723.3	Cervicobrachial syndrome.
	723.4	Brachial neuritis or radiculitis, not otherwise specified
Other digestive diseases	455.1	Internal thrombosed hemorrhoids
	455.4	External thrombosed hemorrhoids
	455.7	Unspecified thrombosed hemorrhoids
	530.1	Esophagitis
	530.12	Acute esophagitis
	530.13	Eosinophilic esophagitis
	530.19	Other esophagitis

© 2020 Stokes A et al. *JAMA Network Open.*

	530.2	Ulcer of esophagus
	530.4	Perforation of esophagus
	530.7	Gastroesophageal laceration-hemorrhage syndrome
	536.3	Gastroparesis
	536.8	Dyspepsia and other specified disorders of function of stomach
	537.3	Other obstruction of duodenum
	538	Gastrointestinal mucositis (ulcerative)
	540	Acute appendicitis
	541	Appendicitis, unqualified
	542	Other appendicitis
	557	Vascular insufficiency of intestine
	560.1	Paralytic ileus
	560.2	Volvulus
	560.89	Other specified intestinal obstruction
	560.9	Unspecified intestinal obstruction
	562.01	Diverticulitis of small intestine (without mention of hemorrhage)
	562.03	Diverticulitis of small intestine with hemorrhage
	562.11	Diverticulitis of colon (without mention of hemorrhage)
	562.13	Diverticulitis of colon with hemorrhage
	569.3	Hemorrhage of rectum and anus
	569.41	Ulcer of anus and rectum
	569.42	Anal or rectal pain
	569.82	Ulceration of intestine
	569.83	Perforation of intestine
	578	Gastrointestinal hemorrhage
	789	Other symptoms involving abdomen and pelvis
	789.6	Abdominal tenderness
	789.7	Colic
	573.4	Hepatic infarction
	577	Diseases of pancreas
	577.1	Other chronic pancreatitis
	577.2	Cyst and pseudocyst of pancreas
	574	Cholelithiasis
	574.1	Calculus of gallbladder with other cholecystitis
	574.3	Calculus of bile duct with acute cholecystitis
	574.4	Calculus of bile duct with other cholecystitis
	574.51	Calculus of bile duct without mention of cholecystitis, with obstruction
	574.7	Calculus of gallbladder and bile duct with other cholecystitis
	574.8	Calculus of gallbladder and bile duct with acute and chronic cholecystitis
	574.91	Calculus of gallbladder and bile duct without cholecystitis, with obstruction
	575	Other disorders of gallbladder
	575.1	Other cholecystitis
	575.12	Acute and chronic cholecystitis
	575.2	Obstruction of gallbladder
	575.3	Hydrops of gallbladder
	575.4	Cholangitis

© 2020 Stokes A et al. *JAMA Network Open.*

	576.1	Cholangitis
	576.2	Obstruction of bile duct
	576.3	Perforation of bile duct
	535	Gastritis and duodenitis
	535.3	Alcoholic gastritis
	535.4	Other specified gastritis
	535.41	Other specified gastritis, with hemorrhage
	535.5	Unspecified gastritis and gastroduodenitis
	535.6	Duodenitis
	535.7	Eosinophilic gastritis
	550.1	Inguinal hernia with obstruction without mention of gangrene
	552	Other hernia of abdominal cavity with obstruction but without mention of gangrene
	558.9	Other and unspecified noninfectious gastroenteritis and colitis
Other injury	800-897	Fracture of skull, spine, trunk, upper or lower limb; dislocation; sprains/strains, intracranial injury, internal injury, open wound of head, neck, trunk, upper or lower limb
	920	Contusion of face, scalp, and neck except eye(s)
	921	Contusion of eye and adnexa
	921.1	Contusion of eyelids and periocular area
	922	Contusion of trunk
	923	Contusion of upper limb
	924	Contusion of lower limb and of other and unspecified sites
	925-949	Crushing injury; effects of foreign body entering through orifice; burns
	953	Injury to nerve roots and spinal plexus
	954.8	Injury to other specified nerve(s) of trunk, excluding shoulder and pelvic girdles
	954.9	Injury to unspecified nerve of trunk excluding shoulder and pelvic girdles
	955-957	Injury to peripheral nerve(s) of shoulder girdle, upper limb, lower limb, pelvic girdle, and other or unspecified nerves
	958.9	Traumatic compartment syndrome
	959	Injury other and unspecified
	997.41	Retained cholelithiasis following cholecystectomy
	997.62	Infection (chronic) of amputation stump
Pain in limb	729.5	Pain in unspecified limb.
Pain in thoracic spine	724.1	Pain in thoracic spine
Peripheral nerve disorder	354	Carpal tunnel syndrome, unspecified upper limb
	355	Mononeuritis of lower limb and unspecified site
	353	Nerve root and plexus disorders
	356	Hereditary and idiopathic peripheral neuropathy
	356.2	Hereditary sensory neuropathy
	356.4	Idiopathic progressive polyneuropathy
	356.8	Other hereditary and idiopathic neuropathies
	356.9	Hereditary and idiopathic neuropathy, unspecified
	357.1	Polyneuropathy in collagen vascular disease
	357.2	Polyneuropathy in diabetes
	357.4	Polyneuropathy in other diseases classified elsewhere
	357.5	Alcoholic polyneuropathy

© 2020 Stokes A et al. *JAMA Network Open.*

	357.7	Polyneuropathy due to other toxic agents
	357.81	Chronic inflammatory demyelinating polyneuritis.
	357.82	Critical illness polyneuropathy
	357.89	Other inflammatory and toxic neuropathy
	357.9	Unspecified inflammatory and toxic neuropathy
	729.2	Neuralgia, neuritis, and radiculitis, unspecified
	350	Trigeminal nerve disorders
Postlaminectomy syndrome or failed back surgery	722.8	Postlaminectomy syndrome
Sciatica	724.3	Sciatica, unspecified side
Thoracic or lumbosacralneuritis or radiculitis	724.4	Thoracic or lumbosacral neuritis or radiculitis, unspecified

© 2020 Stokes A et al. *JAMA Network Open.*

eTable 5. ICD-10 crosswalk from ICD-9 typology to identify pain diagnosis claims

Pain Diagnosis Claims	ICD-10 Codes
Acute pain	G89.11, G89.12, G89.18
Backache	M54.9,M54.08
Cardiovascular and circulatory diseases	I09.2, I26.90, I26.92, I26.99, I30.0, I30.8, I30.9, I32, I40.0, I40.1, I40.8, I40.9, I41, I51.4, I74.2, I74.3, I74.5, I74.8, I74.9, I75.019, I75.029, I75.81, I75.89, I76, I77.6, I80.10, I80.209, I80.219, I80.3, I80.8, I80.9, I82.0, I82.1, I82.409, I82.419, I82.429, I82.439, I82.449, I82.499, I82.4Y9, I82.4Z9, I82.609, I82.629, I82.890, I82.A19, I83.009, I83.019, I83.029, I83.10, I83.209, I83.899, I89.0, I89.1, M35.2, T80.0XX, T81.718, T81.72X, T82.817, T82.818,R07.1, R07.2, R07.81, R07.82, R07.89, R07.9
Cervicalgia	M43.6, M54.2
Chronic pain	G89.21, G89.22, G89.28, G89.29,G89.4
Degeneration of intervertebral disc	M50.30, M51.34, M51.35, M51.36, M51.37
Diseases of connective tissue	M32.10, M34.0, M34.1, M34.9, M54.02, M66.239, M66.249, M66.259, M66.269, M66.339, M66.349, M66.369, M66.829, M66.879, M66.88, M66.9, M70.039, M70.30, M70.40, M71.50, M72.9, M75.120, M77.40, M79.3, M79.4
Disorder of joint	M02.30, M02.00, M02.20, M02.9, M12.10, M12.119, M12.129, M12.139, M12.149, M12.159, M12.169, M12.179, M12.18, M12.19, M12.20, M12.219, M12.229, M12.239, M12.249, M12.259, M12.269, M12.279, M12.28, M12.29, M12.30, M12.319, M12.329, M12.339, M12.349, M12.359, M12.369, M12.379, M12.38, M12.39, M12.40, M12.419, M12.429, M12.439, M12.449, M12.459, M12.469, M12.479, M12.48, M12.49, M12.50, M12.519, M12.529, M12.539, M12.549, M12.559, M12.569, M12.579, M12.58, M12.59, M12.80, M12.819, M12.829, M12.839, M12.849, M12.859, M12.869, M12.879, M12.88, M12.89, M12.9, M13.0, M13.10, M13.119, M13.129, M13.139, M13.149, M13.159, M13.169, M13.179, M13.80, M13.819, M13.829, M13.839, M13.849, M13.859, M13.869, M13.879, M13.88, M13.89, M14.60, M14.80, M22.40, M23.009, M23.202, M23.205, M23.219, M23.229, M23.239, M23.249, M23.259, M23.269, M23.305, M23.319, M23.329, M23.339, M23.349, M23.359, M23.369, M23.40, M23.50, M23.8X9, M23.90, M24.00, M24.019, M24.029, M24.039, M24.049, M24.059, M24.073, M24.076, M24.08, M24.10, M24.119, M24.129, M24.139, M24.149, M24.159, M24.173, M24.176, M24.30, M24.319, M24.329, M24.339, M24.349, M24.359, M24.369, M24.373, M24.376, M24.40, M24.419, M24.429, M24.439, M24.443, M24.446, M24.459, M24.469, M24.473, M24.476, M24.80, M24.819, M24.829, M24.839, M24.849, M24.859, M24.873, M24.876, M24.9, M25.00, M25.019, M25.029, M25.039, M25.049, M25.059, M25.069, M25.073, M25.076, M25.08, M25.50, M25.519, M25.529, M25.539, M25.541, M25.542, M25.549, M25.559, M25.569, M25.579, M36.2, M36.3, M36.4, M43.4, M43.5X9, M79.0, M79.646 M05.00, M05.30, M05.60, M06.1, M06.4, M06.9, M08.00, M08.3, M08.40, M12.00, L40.54, L40.59, M45.9, M46.00, M46.1, M46.80, M46.90, M49.80 M10.00, M10.9, M11.20, M11.219, M11.229, M11.239, M11.249, M11.259, M11.269, M11.279, M11.28, M11.29, M11.80, M11.819, M11.829, M11.839, M11.849, M11.859, M11.869, M11.879, M11.88, M11.89, M11.9, M1A.00X1, M1A.20X1, M1A.30X1, M1A.40X1, M1A.9XX0, M1A.9XX1
Displacement of intervertebral disc	M50.20, M51.24, M51.25, M51.26, M51.27, M51.44, M51.45, M51.46, M51.47, M51.9
Generalized pain, symptoms	R52
Headache syndromes	G44.009, G44.019, G44.029, G44.039, G44.049, G44.059, G44.099, G44.1, G44.209, G44.219, G44.221, G44.229, G44.309, G44.319, G44.329, G44.41, G44.51, G44.52, G44.53, G44.59, G44.81, G44.82, G44.83, G44.84, G44.85, G44.89, R51,G43.001, G43.009, G43.011, G43.019, G43.101, G43.109, G43.111, G43.119, G43.401, G43.409, G43.411, G43.419, G43.501, G43.509, G43.511, G43.519, G43.601, G43.609, G43.611, G43.619, G43.701, G43.709, G43.711, G43.719, G43.801, G43.809, G43.811, G43.819, G43.821, G43.829, G43.831, G43.839, G43.901, G43.909, G43.911, G43.919, G43.A0, G43.A1, G43.B0, G43.B1, G43.C0, G43.C1, G43.D0, G43.D1
Myopathy	G72.2, G72.41, G72.49, G72.81, G72.89, G72.9, G73.7, M33.03, M33.13, M33.20, M33.90, M33.93, M35.3, M35.8, M60.10, M60.9, M61.00, M61.10, M61.40, M61.59, M61.9, M62.10, M62.40, M62.82, M62.838, M79.1, M79.7, M79.A19, M79.A29, M79.A3, M79.A9, R25.2
Osteoarthritis	M15.0, M15.1, M15.2, M15.3, M15.8, M15.9, M16.10, M16.7, M16.9, M17.10, M17.5, M17.9, M18.9, M19.019, M19.029, M19.039, M19.049, M19.079, M19.219, M19.229, M19.239, M19.249, M19.279, M19.90, M19.91, M19.93
Other back disorders	M54.5, M43.8X9, M53.9, M47.10, M47.12, M47.14, M47.16, M47.812, M47.814, M47.817, M47.819, M48.10, M48.20, M48.30, M48.9
Other cervical region disorders	M53.0, M53.1, M54.12
Other digestive diseases	K20.0, K20.8, K20.9, K22.10, K22.11, K22.3, K22.6, K30, K31.5, K31.84, K35.2, K35.3, K35.80, K35.89, K36, K37, K55.011, K55.012, K55.019, K55.021, K55.022, K55.029, K55.031, K55.032, K55.039, K55.041, K55.042, K55.049, K55.051, K55.052, K55.059, K55.061, K55.062, K55.069, K55.30, K55.31, K55.32, K55.33, K56.0, K56.2, K56.600, K56.601, K56.609, K56.690, K56.691, K56.699, K56.7, K57.12, K57.13, K57.32, K57.33, K62.5, K62.6, K62.89, K63.1, K63.3, K64.5, K64.8, K92.0, K92.1, K92.2, K92.81, R10.10, R10.11, R10.12, R10.13, R10.30, R10.31, R10.32, R10.33, R10.811, R10.812, R10.813, R10.814, R10.815, R10.816, R10.817, R10.819, R10.821, R10.822, R10.823, R10.824, R10.825, R10.826, R10.827, R10.829, R10.83, R10.84, R10.9, K76.3, K85.90, K85.91, K85.92, K86.1, K86.2, K86.3, K80.00, K80.01, K80.18, K80.19, K80.42, K80.43, K80.44, K80.45, K80.51, K80.64, K80.65, K80.66, K80.67, K80.71, K81.0, K81.2, K81.9, K82.0, K82.1, K82.2, K83.0, K83.1, K83.2, K29.00, K29.01, K29.20, K29.21, K29.60, K29.61, K29.70, K29.71, K29.80, K29.81, K29.90, K29.91, K40.30, K40.31, K40.00, K40.01, K52.3, K52.89, K52.9, K41.00, K41.01, K41.30, K41.31, K42.0, K43.0, K43.6, K44.0, K45.0, K46.0

© 2020 Stokes A et al. *JAMA Network Open.*

Other injury	K91.86, S00.10XA, S00.93XA, S01.00XA, S01.02XA, S01.109A, S01.119A, S01.129A, S01.139A, S01.149A, S01.159A, S01.20XA, S01.21XA, S01.22XA, S01.23XA, S01.24XA, S01.25XA, S01.309A, S01.329A, S01.409A, S01.422A, S01.429A, S01.501A, S01.502A, S01.512A, S01.521A, S01.522A, S01.80XA, S01.82XA, S01.90XA, S01.92XA, S02.0XXA, S02.0XXB, S02.101A, S02.101B, S02.102A, S02.102B, S02.109A, S02.109B, S02.2XXA, S02.2XXB, S02.30XA, S02.30XB, S02.31XA, S02.31XB, S02.32XA, S02.32XB, S02.400A, S02.400B, S02.401A, S02.401B, S02.402A, S02.402B, S02.40AA, S02.40AB, S02.40BA, S02.40BB, S02.40CA, S02.40CB, S02.40DA, S02.40DB, S02.40EA, S02.40EB, S02.40FA, S02.40FB, S02.42XA, S02.42XB, S02.5XXA, S02.5XXB, S02.600A, S02.600B, S02.601A, S02.601B, S02.602A, S02.602B, S02.609A, S02.609B, S02.610A, S02.610B, S02.611A, S02.611B, S02.612A, S02.612B, S02.620A, S02.620B, S02.621A, S02.621B, S02.622A, S02.622B, S02.630A, S02.630B, S02.631A, S02.631B, S02.632A, S02.632B, S02.640A, S02.640B, S02.641A, S02.641B, S02.642A, S02.642B, S02.650A, S02.650B, S02.651A, S02.651B, S02.652A, S02.652B, S02.66XA, S02.66XB, S02.670A, S02.670B, S02.671A, S02.671B, S02.672A, S02.672B, S02.69XA, S02.69XB, S02.91XA, S02.91XB, S02.92XA, S02.92XB, S03.00XA, S03.01XA, S03.02XA, S03.03XA, S03.1XXA, S03.40XA, S03.41XA, S03.42XA, S03.43XA, S03.8XXA, S03.9XXA, S05.20XA, S05.30XA, S05.40XA, S05.50XA, S05.60XA, S05.70XA, S05.90XA, S06.0X0A, S06.0X1A, S06.0X9A, S06.1X0A, S06.1X1A, S06.1X2A, S06.1X3A, S06.1X4A, S06.1X5A, S06.1X6A, S06.1X7A, S06.1X8A, S06.1X9A, S06.330A, S06.331A, S06.332A, S06.333A, S06.334A, S06.335A, S06.336A, S06.337A, S06.338A, S06.339A, S06.360A, S06.361A, S06.362A, S06.363A, S06.364A, S06.365A, S06.366A, S06.367A, S06.368A, S06.369A, S06.370A, S06.371A, S06.372A, S06.373A, S06.374A, S06.375A, S06.376A, S06.377A, S06.378A, S06.379A, S06.380A, S06.381A, S06.382A, S06.383A, S06.384A, S06.385A, S06.386A, S06.387A, S06.388A, S06.389A, S06.4X0A, S06.4X1A, S06.4X2A, S06.4X3A, S06.4X4A, S06.4X5A, S06.4X6A, S06.4X7A, S06.4X8A, S06.4X9A, S06.5X0A, S06.5X1A, S06.5X2A, S06.5X3A, S06.5X4A, S06.5X5A, S06.5X6A, S06.5X7A, S06.5X8A, S06.5X9A, S06.6X0A, S06.6X1A, S06.6X2A, S06.6X3A, S06.6X4A, S06.6X5A, S06.6X6A, S06.6X7A, S06.6X8A, S06.6X9A, S06.890A, S06.891A, S06.892A, S06.893A, S06.894A, S06.895A, S06.896A, S06.897A, S06.898A, S06.899A, S06.9X0A, S06.9X1A, S06.9X2A, S06.9X3A, S06.9X4A, S06.9X5A, S06.9X6A, S06.9X7A, S06.9X8A, S06.9X9A, S07.0XXA, S07.8XXA, S08.119A, S08.129A, S08.811A, S09.20XA, S09.309A, S09.399A, S09.8XXA, S09.90XA, S09.91XA, S09.93XA, S10.93XA, S11.012A, S11.014A, S11.019A, S11.022A, S11.024A, S11.029A, S11.10XA, S11.12XA, S11.20XA, S11.22XA, S11.80XA, S11.82XA, S11.90XA, S12.000A, S12.000B, S12.001A, S12.001B, S12.100A, S12.100B, S12.101A, S12.101B, S12.200A, S12.200B, S12.201A, S12.201B, S12.300A, S12.300B, S12.301A, S12.301B, S12.400A, S12.400B, S12.401A, S12.401B, S12.500A, S12.500B, S12.501A, S12.501B, S12.600A, S12.600B, S12.601A, S12.601B, S12.8XXA, S12.9XXA, S13.101A, S13.111A, S13.121A, S13.131A, S13.141A, S13.151A, S13.161A, S13.171A, S13.181A, S13.4XXA, S13.5XXA, S13.8XXA, S14.101A, S14.102A, S14.103A, S14.104A, S14.105A, S14.106A, S14.107A, S14.109A, S14.111A, S14.112A, S14.113A, S14.114A, S14.115A, S14.116A, S14.117A, S14.121A, S14.122A, S14.123A, S14.124A, S14.125A, S14.126A, S14.127A, S14.131A, S14.132A, S14.133A, S14.134A, S14.135A, S14.136A, S14.137A, S14.151A, S14.152A, S14.153A, S14.154A, S14.155A, S14.156A, S14.157A, S14.2XXA, S14.3XXA, S14.4XXA, S14.9XXA, S17.9XXA, S19.9XXA, S20.00XA, S20.20XA, S20.219A, S20.229A, S21.009A, S21.029A, S21.109A, S21.129A, S21.209A, S21.309A, S22.009A, S22.009B, S22.019A, S22.019B, S22.029A, S22.029B, S22.039A, S22.039B, S22.049A, S22.049B, S22.059A, S22.059B, S22.069A, S22.069B, S22.079A, S22.079B, S22.089A, S22.089B, S22.20XA, S22.20XB, S22.39XA, S22.39XB, S22.49XA, S22.49XB, S22.5XXA, S22.5XXB, S22.9XXA, S22.9XXB, S23.101A, S23.20XA, S23.3XXA, S23.41XA, S23.420A, S23.421A, S23.428A, S23.429A, S23.8XXA, S23.9XXA, S24.101A, S24.102A, S24.103A, S24.104A, S24.109A, S24.111A, S24.112A, S24.113A, S24.114A, S24.131A, S24.132A, S24.133A, S24.134A, S24.151A, S24.152A, S24.153A, S24.154A, S24.2XXA, S24.3XXA, S24.8XXA, S24.9XXA, S26.020A, S26.021A, S26.022A, S26.10XA, S26.90XA, S26.91XA, S26.92XA, S26.99XA, S27.0XXA, S27.1XXA, S27.2XXA, S27.309A, S27.329A, S27.339A, S27.409A, S27.809A, S27.813A, S27.819A, S27.893A, S27.899A, S27.9XXA, S28.0XXA, S29.019A, S29.8XXA, S30.0XXA, S30.1XXA, S30.201A, S30.202A, S31.000A, S31.001A, S31.020A, S31.102A, S31.105A, S31.109A, S31.122A, S31.125A, S31.129A, S31.20XA, S31.22XA, S31.30XA, S31.32XA, S31.40XA, S31.42XA, S31.501A, S31.502A, S31.522A, S31.541A, S31.609A, S31.802A, S31.809A, S32.009A, S32.009B, S32.019A, S32.019B, S32.029A, S32.029B, S32.039A, S32.039B, S32.049A, S32.049B, S32.059A, S32.059B, S32.10XA, S32.10XB, S32.2XXA, S32.2XXB, S32.309A, S32.309B, S32.409A, S32.409B, S32.501A, S32.501B, S32.502A, S32.502B, S32.509A, S32.509B, S32.609A, S32.609B, S32.810A, S32.810B, S32.811A, S32.811B, S32.82XA, S32.82XB, S32.89XA, S32.89XB, S32.9XXA, S32.9XXB, S33.101A, S33.2XXA, S33.39XA, S33.5XXA, S33.6XXA, S33.8XXA, S33.9XXA, S34.101A, S34.102A, S34.103A, S34.104A, S34.105A, S34.109A, S34.111A, S34.112A, S34.113A, S34.114A, S34.115A, S34.119A, S34.121A, S34.122A, S34.123A, S34.124A, S34.125A, S34.129A, S34.131A, S34.132A, S34.139A, S34.21XA, S34.22XA, S34.3XXA, S34.4XXA, S34.6XXA, S34.9XXA, S36.00XA, S36.020A, S36.021A, S36.029A, S36.030A, S36.031A, S36.032A, S36.09XA, S36.112A, S36.113A, S36.114A, S36.115A, S36.116A, S36.118A, S36.119A, S36.129A, S36.13XA, S36.200A, S36.201A, S36.202A, S36.209A, S36.30XA, S36.400A, S36.408A, S36.409A, S36.500A, S36.501A, S36.502A, S36.503A, S36.508A, S36.509A, S36.60XA, S36.81XA, S36.899A, S36.90XA, S37.009A, S37.019A, S37.029A, S37.039A, S37.049A, S37.059A, S37.069A, S37.10XA, S37.20XA, S37.30XA, S37.409A, S37.509A, S37.60XA, S37.819A, S37.829A, S37.899A, S37.90XA, S38.001A, S38.002A, S38.1XXA, S38.221A, S38.222A, S39.011A, S39.81XA, S39.82XA, S39.840A, S39.848A, S40.019A, S40.029A, S41.009A, S41.029A, S41.109A, S41.129A, S42.009A, S42.009B, S42.013A, S42.013B, S42.016A, S42.016B, S42.019A, S42.019B, S42.023A, S42.023B, S42.026A, S42.026B, S42.033A, S42.033B, S42.036A, S42.036B, S42.109A, S42.109B, S42.113A, S42.113B, S42.116A, S42.116B, S42.123A, S42.123B, S42.126A, S42.126B, S42.133A, S42.133B, S42.136A, S42.136B, S42.143A, S42.143B, S42.146A, S42.146B, S42.153A, S42.153B, S42.156A, S42.156B, S42.199A, S42.199B, S42.209A, S42.209B, S42.213A, S42.213B, S42.216A, S42.216B, S42.253A, S42.253B, S42.256A, S42.256B, S42.293A, S42.293B, S42.295A, S42.296A, S42.296B, S42.309A, S42.309B, S42.399A, S42.399B, S42.409A, S42.409B, S42.413A, S42.413B, S42.416A, S42.416B, S42.433A, S42.436A, S42.443A, S42.446A, S42.453A, S42.453B, S42.456A, S42.456B, S42.463A, S42.463B, S42.466A, S42.466B, S42.473A, S42.473B, S42.476A, S42.476B, S42.493A, S42.493B, S42.496A, S42.496B, S42.90XA, S42.90XB, S42.91XA, S42.91XB, S42.92XA, S42.92XB, S43.006A, S43.016A, S43.026A, S43.036A, S43.086A, S43.109A, S43.206A, S43.409A, S43.419A, S43.429A, S43.439A, S43.499A, S43.50XA, S43.80XA, S44.00XA, S44.10XA, S44.20XA, S44.30XA, S44.40XA, S44.50XA, S44.8X9A, S44.90XA, S46.019A, S46.119A, S46.819A, S46.919A, S46.929A, S47.9XXA, S48.019A, S48.029A, S48.119A, S48.129A, S48.911A, S48.912A, S48.919A, S48.921A, S48.922A, S48.929A, S49.80XA, S49.90XA, S50.00XA, S50.10XA, S51.009A, S51.029A, S51.809A, S51.829A, S52.009A, S52.009B, S52.009C, S52.011A, S52.012A, S52.019A, S52.023A, S52.023B, S52.023C, S52.026A, S52.026B, S52.026C

© 2020 Stokes A et al. *JAMA Network Open.*

Other injury (cont.)	S52.043A, S52.043B, S52.043C, S52.046A, S52.046B, S52.046C, S52.099A, S52.099B, S52.099C, S52.109A, S52.109B, S52.109C, S52.111A, S52.112A, S52.119A, S52.123A, S52.123B, S52.123C, S52.126A, S52.126B, S52.126C, S52.133A, S52.133B, S52.133C, S52.136A, S52.136B, S52.136C, S52.189A, S52.189B, S52.189C, S52.209A, S52.209B, S52.209C, S52.279A, S52.279B, S52.279C, S52.309A, S52.309B, S52.309C, S52.509A, S52.509B, S52.509C, S52.521A, S52.522A, S52.529A, S52.539A, S52.539B, S52.539C, S52.549A, S52.609A, S52.609B, S52.609C, S52.621A, S52.622A, S52.629A, S52.90XA, S52.90XB, S52.90XC, S52.91XA, S52.91XB, S52.92XA, S52.92XB, S53.006A, S53.016A, S53.026A, S53.033A, S53.096A, S53.106A, S53.116A, S53.136A, S53.146A, S53.196A, S53.409A, S53.419A, S53.429A, S53.439A, S53.449A, S53.499A, S54.00XA, S54.10XA, S54.20XA, S54.30XA, S56.919A, S56.929A, S57.00XA, S57.80XA, S58.019A, S58.029A, S58.119A, S58.129A, S58.919A, S58.929A, S59.109A, S59.809A, S59.819A, S59.909A, S59.919A, S60.00XA, S60.019A, S60.10XA, S60.219A, S60.229A, S61.009A, S61.109A, S61.209A, S61.229A, S61.409A, S61.429A, S61.509A, S61.529A, S62.009A, S62.009B, S62.109A, S62.109B, S62.113A, S62.113B, S62.116A, S62.116B, S62.123A, S62.123B, S62.126A, S62.126B, S62.133A, S62.133B, S62.136A, S62.136B, S62.143A, S62.143B, S62.146A, S62.146B, S62.153A, S62.153B, S62.156A, S62.156B, S62.163A, S62.163B, S62.166A, S62.166B, S62.173A, S62.173B, S62.176A, S62.176B, S62.183A, S62.183B, S62.186A, S62.186B, S62.233A, S62.233B, S62.236A, S62.236B, S62.309A, S62.309B, S62.319A, S62.319B, S62.329A, S62.329B, S62.339A, S62.339B, S62.349A, S62.349B, S62.359A, S62.359B, S62.369A, S62.369B, S62.399A, S62.399B, S62.509A, S62.509B, S62.513A, S62.513B, S62.516A, S62.516B, S62.523A, S62.523B, S62.526A, S62.526B, S62.609A, S62.609B, S62.619B, S62.629A, S62.629B, S62.639A, S62.639B, S62.649A, S62.649B, S62.659A, S62.659B, S62.669A, S62.669B, S62.90XA, S62.90XB, S63.006A, S82.856C, S82.899A, S82.899B, S82.899C, S82.90XA, S82.90XB, S82.91XA, S82.91XB, S82.92XA, S82.92XB, S83.006A, S83.106A, S83.116A, S83.126A, S83.136A, S83.146A, S83.196A, S83.209A, S83.219A, S83.249A, S83.289A, S83.30XA, S83.419A, S83.429A, S83.509A, S83.60XA, S83.8X9A, S83.90XA, S84.00XA, S84.10XA, S84.20XA, S84.809A, S84.90XA, S86.019A, S86.819A, S86.919A, S86.929A, S87.00XA, S87.80XA, S88.019A, S88.029A, S88.119A, S88.129A, S88.911A, S88.912A, S88.919A, S88.929A, S89.80XA, S89.90XA, S90.00XA, S90.119A, S90.129A, S90.229A, S90.30XA, S91.009A, S91.029A, S91.109A, S91.129A, S91.209A, S91.309A, S91.329A, S92.009A, S92.009B, S92.109A, S92.109B, S92.201A, S92.201B, S92.202A, S92.202B, S92.209A, S92.209B, S92.213A, S92.213B, S92.216A, S92.216B, S92.223A, S92.223B, S92.226A, S92.226B, S92.233A, S92.233B, S92.236A, S92.236B, S92.243A, S92.243B, S92.246A, S92.246B, S92.253A, S92.253B, S92.256A, S92.256B, S92.301A, S92.301B, S92.302A, S92.302B, S92.309A, S92.309B, S92.403A, S92.403B, S92.406A, S92.406B, S92.503A, S92.503B, S92.506A, S92.506B, S92.819A, S92.819B, S92.909A, S92.909B, S93.06XA, S93.106A, S93.119A, S93.129A, S93.306A, S93.316A, S93.326A, S93.336A, S93.409A, S93.419A, S93.429A, S93.439A, S93.499A, S93.519A, S93.529A, S93.609A, S93.629A, S93.699A, S94.30XA, S94.8X9A, S94.90XA, S96.919A, S96.929A, S97.00XA, S97.109A, S97.80XA, S98.019A, S98.029A, S98.119A, S98.129A, S98.139A, S98.149A, S98.219A, S98.229A, S98.319A, S98.329A, S98.911A, S98.912A, S98.919A, S98.921A, S98.922A, S98.929A, S99.009A, S99.009B, S99.019A, S99.019B, S99.029A, S99.029B, S99.039A, S99.039B, S99.049A, S99.049B, S99.099A, S99.099B, S99.109A, S99.109B, S99.119A, S99.119B, S99.129A, S99.129B, S99.139A, S99.139B, S99.149A, S99.149B, S99.199A, S99.199B, S99.819A, S99.919A, T07.XXXA, T14.8XXA, T14.90XA, T15.00XA, T15.01XA, T15.02XA, T15.10XA, T15.11XA, T15.12XA, T15.80XA, T15.81XA, T15.82XA, T15.90XA, T15.91XA, T15.92XA, T16.1XXA, T16.2XXA, T16.9XXA, T17.0XXA, T17.1XXA, T17.200A, T17.208A, T17.210A, T17.218A, T17.220A, T17.228A, T17.290A, T17.298A, T17.300A, T17.308A, T17.310A, T17.318A, T17.320A, T17.328A, T17.390A, T17.398A, T17.400A, T17.408A, T17.410A, T17.418A, T17.420A, T17.428A, T17.490A, T17.498A, T17.500A, T17.508A, T17.510A, T17.518A, T17.520A, T17.528A, T17.590A, T17.598A, T17.800A, T17.808A, T17.810A, T17.818A, T17.820A, T17.828A, T17.890A, T17.898A, T17.900A, T17.908A, T17.910A, T17.918A, T17.920A, T17.928A, T17.990A, T17.998A, T18.0XXA, T18.100A, T18.108A, T18.110A, T18.118A, T18.120A, T18.128A, T18.190A, T18.198A, T18.2XXA, T18.3XXA, T18.4XXA, T18.5XXA, T18.8XXA, T18.9XXA, T19.0XXA, T19.1XXA, T19.2XXA, T19.3XXA, T19.4XXA, T19.8XXA, T19.9XXA, T20.00XA, T20.019A, T20.02XA, T20.03XA, T20.04XA, T20.05XA, T20.06XA, T20.07XA, T20.09XA, T20.10XA, T20.119A, T20.12XA, T20.13XA, T20.14XA, T20.15XA, T20.16XA, T20.17XA, T20.19XA, T20.20XA, T20.219A, T20.22XA, T20.23XA, T20.24XA, T20.25XA, T20.26XA, T20.27XA, T20.29XA, T20.30XA, T20.319A, T20.32XA, T20.33XA, T20.34XA, T20.35XA, T20.36XA, T20.37XA, T20.39XA, T20.40XA, T20.419A, T20.42XA, T20.43XA, T20.44XA, T20.45XA, T20.46XA, T20.47XA, T20.49XA, T20.50XA, T20.519A, T20.52XA, T20.53XA, T20.54XA, T20.55XA, T20.56XA, T20.57XA, T20.59XA, T20.60XA, T20.619A, T20.62XA, T20.63XA, T20.64XA, T20.65XA, T20.66XA, T20.67XA, T20.69XA, T20.70XA, T20.719A, T20.72XA, T20.73XA, T20.74XA, T20.75XA, T20.76XA, T20.77XA, T20.79XA, T21.00XA, T21.01XA, T21.02XA, T21.03XA, T21.06XA, T21.07XA, T21.09XA, T21.10XA, T21.11XA, T21.12XA, T21.13XA, T21.16XA, T21.17XA, T21.19XA, T21.20XA, T21.21XA, T21.22XA, T21.23XA, T21.26XA, T21.27XA, T21.29XA, T21.30XA, T21.31XA, T21.32XA, T21.33XA, T21.36XA, T21.37XA, T21.39XA, T21.40XA, T21.41XA, T21.42XA, T21.43XA, T21.46XA, T21.47XA, T21.49XA, T21.50XA, T21.51XA, T21.52XA, T21.53XA, T21.56XA, T21.57XA, T21.59XA, T21.60XA, T21.61XA, T21.62XA, T21.63XA, T21.66XA, T21.67XA, T21.69XA, T21.70XA, T21.71XA, T21.72XA, T21.73XA, T21.76XA, T21.77XA, T21.79XA, T22.00XA, T22.019A, T22.029A, T22.039A, T22.049A, T22.059A, T22.069A, T22.099A, T22.10XA, T22.119A, T22.129A, T22.139A, T22.149A, T22.159A, T22.169A, T22.199A, T22.20XA, T22.219A, T22.229A, T22.239A, T22.249A, T22.259A, T22.269A, T22.299A, T22.30XA, T22.319A, T22.329A, T22.339A, T22.349A, T22.359A, T22.369A, T22.399A, T22.40XA, T22.419A, T22.429A, T22.439A, T22.449A, T22.459A, T22.469A, T22.499A, T22.50XA, T22.519A, T22.529A, T22.539A, T22.549A, T22.559A, T22.569A, T22.599A, T22.60XA, T22.619A, T22.629A, T22.639A, T22.649A, T22.659A, T22.669A, T22.699A, T22.70XA, T22.719A, T22.729A, T22.739A, T22.749A, T22.759A, T22.769A, T22.799A, T23.009A, T23.019A, T23.029A, T23.039A, T23.049A, T23.059A, T23.069A, T23.079A, T23.099A, T23.109A, T23.119A, T23.129A, T23.139A, T23.149A, T23.159A, T23.169A, T23.179A, T23.199A, T23.209A, T23.219A, T23.229A, T23.239A, T23.249A, T23.259A, T23.269A, T23.279A, T23.299A, T23.309A, T23.319A, T23.329A, T23.339A, T23.349A, T23.359A, T23.369A, T23.379A, T23.399A, T23.409A, T23.419A, T23.429A, T23.439A, T23.449A, T23.459A, T23.469A, T23.479A, T23.499A, T23.509A, T23.519A, T23.529A, T23.539A, T23.549A, T23.559A, T23.569A, T23.579A, T23.599A, T23.609A, T23.619A, T23.629A, T23.639A, T23.649A, T23.659A, T23.669A, T23.679A, T23.699A, T23.709A, T23.719A, T23.729A, T23.739A, T23.749A, T23.759A, T23.769A, T23.779A, T23.799A, T24.009A, T24.019A, T24.029A, T24.039A, T24.099A, T24.109A, T24.119A, T24.129A, T24.139A, T24.199A, T24.209A, T24.219A, T24.229A, T24.239A, T24.299A, T24.309A, T24.319A, T24.329A, T24.339A, T24.399A, T24.409A, T24.419A, T24.429A, T24.439A, T24.499A, T24.509A, T24.519A, T24.529A, T24.539A, T24.599A, T24.609A, T24.619A, T24.629A, T24.639A, T24.699A, T24.709A, T24.719A, T24.729A, T24.739A, T24.799A, T25.019A, T25.029A, T25.039A, T25.099A, T25.119A, T25.129A, T25.139A, T25.199A, T25.219A, T25.229A, T25.239A, T25.299A, T25.319A, T25.329A, T25.339A, T25.399A,

© 2020 Stokes A et al. *JAMA Network Open.*

Other injury (cont.)	T25.419A, T25.429A, T25.439A, T25.499A, T25.519A, T25.529A, T25.539A, T25.599A, T25.619A, T25.629A, T25.639A, T25.699A, T25.719A, T25.729A, T25.739A, T25.799A, T26.00XA, T26.10XA, T26.20XA, T26.21XA, T26.22XA, T26.40XA, T26.41XA, T26.42XA, T26.50XA, T26.60XA, T26.70XA, T26.90XA, T27.1XXA, T27.5XXA, T28.0XXA, T28.1XXA, T28.2XXA, T28.3XXA, T28.40XA, T28.49XA, T28.5XXA, T28.6XXA, T28.7XXA, T28.8XXA, T28.90XA, T28.99XA, T30.0, T30.4, T31.0, T31.10, T31.11, T31.20, T31.21, T31.22, T31.30, T31.31, T31.32, T31.33, T31.40, T31.41, T31.42, T31.43, T31.44, T31.50, T31.51, T31.52, T31.53, T31.54, T31.55, T31.60, T31.61, T31.62, T31.63, T31.64, T31.65, T31.66, T31.70, T31.71, T31.72, T31.73, T31.74, T31.75, T31.76, T31.77, T31.80, T31.81, T31.82, T31.83, T31.84, T31.85, T31.86, T31.87, T31.88, T31.90, T31.91, T31.92, T31.93, T31.94, T31.95, T31.96, T31.97, T31.98, T31.99, T32.0, T32.10, T32.11, T32.20, T32.21, T32.22, T32.30, T32.31, T32.32, T32.33, T32.40, T32.41, T32.42, T32.43, T32.44, T32.50, T32.51, T32.52, T32.53, T32.54, T32.55, T32.60, T32.61, T32.62, T32.63, T32.64, T32.65, T32.66, T32.70, T32.71, T32.72, T32.73, T32.74, T32.75, T32.76, T32.77, T32.80, T32.81, T32.82, T32.83, T32.84, T32.85, T32.86, T32.87, T32.88, T32.90, T32.91, T32.92, T32.93, T32.94, T32.95, T32.96, T32.97, T32.98, T32.99, T79.A0XA, T79.A19A, T79.A29A, T79.A3XA, T79.A9XA, T87.40, L10.0, L10.1, L10.2, L10.4, L10.9, L52, L73.2, L89.009, L89.119, L89.129, L89.139, L89.149, L89.159, L89.209, L89.309, L89.509, L89.609, L89.819, L89.899, L89.90, L97.109, L97.209, L97.309, L97.409, L97.509, L97.809, L97.909, L98.419, L98.429, L98.499
Pain in limb	M79.609
Pain in thoracic spine	M54.6
Peripheral nerve disorder	E10.42, E11.42, G54.0, G54.1, G54.2, G54.3, G54.4, G54.5, G54.6, G54.7, G54.8, G54.9, G56.00, G56.10, G56.20, G56.30, G56.40, G56.80, G56.90, G57.00, G57.10, G57.20, G57.30, G57.40, G57.50, G57.60, G57.70, G57.80, G57.90, G58.7, G58.9, G60.0, G60.3, G60.8, G60.9, G61.81, G61.82, G61.89, G61.9, G62.1, G62.2, G62.81, G63, M54.10, M79.2, G50.0, G50.1, G50.8, G50.9
Postlaminectomy syndrome or failed back surgery	M96.1
Sciatica	M54.30
Thoracic or lumbosacralneuritis or radiculitis	M54.14, M54.15, M54.16, M54.17

© 2020 Stokes A et al. *JAMA Network Open.*
